# An archosauromorph dominated ichnoassemblage in fluvial settings from the late Early Triassic of the Catalan Pyrenees (NE Iberian Peninsula)

**DOI:** 10.1371/journal.pone.0174693

**Published:** 2017-04-19

**Authors:** Eudald Mujal, Josep Fortuny, Arnau Bolet, Oriol Oms, José Ángel López

**Affiliations:** 1 Departament de Geologia, Universitat Autònoma de Barcelona, Bellaterra, Spain; 2 Centre de Recherches en Paléobiodiversité et Paléoenvironnements, Muséum National d’Histoire Naturelle, Bâtiment de Paléonologie, Paris, France; 3 Institut Català de Paleontologia Miquel Crusafont, ICTA-ICP Building, Cerdanyola del Vallès, Spain; University of Utah, UNITED STATES

## Abstract

The vertebrate recovery after the end-Permian mass extinction can be approached through the ichnological record, which is much more abundant than body fossils. The late Olenekian (Early Triassic) tetrapod ichnoassemblage of the Catalan Pyrenean Basin is the most complete and diverse of this age from Western Tethys. This extensional basin, composed of several depocenters, was formed in the latest phases of the Variscan orogeny (Pangea breakup) and was infilled by braided and meandering fluvial systems of the red-beds Buntsandstein facies. Abundant and diverse tetrapod ichnites are recorded in these facies, including *Prorotodactylus mesaxonichnus* isp. nov. (tracks possibly produced by euparkeriids), cf. *Rotodactylus*, at least two large chirotheriid morphotypes (archosauriform trackmakers), *Rhynchosauroides* cf. *schochardti*, two other undetermined *Rhynchosauroides* forms, an undetermined Morphotype A (archosauromorph trackmakers) and two types of *Characichnos* isp. (swimming traces, here associated to archosauromorph trackmakers). The Pyrenean ichnoassemblage suggests a relatively homogeneous ichnofaunal composition through the late Early Triassic of Central Pangea, characterized by the presence of *Prorotodactylus* and *Rotodactylus*. Small archosauromorph tracks dominate and present a wide distribution through the different fluviatile facies of the Triassic Pyrenean Basin, with large archosaurian footprints being present in a lesser degree. Archosauromorphs radiated and diversified through the Triassic vertebrate recovery, which ultimately lead to the archosaur and dinosaur dominance of the Mesozoic.

## Introduction

The earliest Mesozoic fossil record represents the life recovery and ecosystems turnover [1] after the loss of most species during the end-Permian mass extinction [2]. The environmental and climatic conditions hindered recovery of life on land, delaying the recovery until the Middle Triassic [3, 4]. Concerning the vertebrate non-marine fauna, several lineage pulses took place during the Early Triassic [4], but the role of the main tetrapod groups in the recovery, as well as their paleogeographic and environmental distribution, are still a matter of discussion.

The earliest Triassic non-marine tetrapod record (Induan to middle Olenekian) is only known from a few areas, such as the South-African Karoo Basin [5] and the Russian South Urals [1]. Most of the Early Triassic record is restricted to the late Olenekian, as occurs in the Western Tethys basins, mainly consisting of red-bed deposits of complex alluvial and aeolian continental systems [6–8]. The few Western Tethys vertebrate records, mostly footprints, are known from Morocco [9, 10], the Italian Southern Alps [11], southern Austria (the only known early Olenekian locality) [12], and the Catalan Pyrenees [8]. Further Lower Triassic records are found in the well-known archosaur-dominated tracksites from the Central European Germanic Basin [13–19], and in the Red Peak and Moenkopi formations of the USA [20–22].

**Fig 1:**
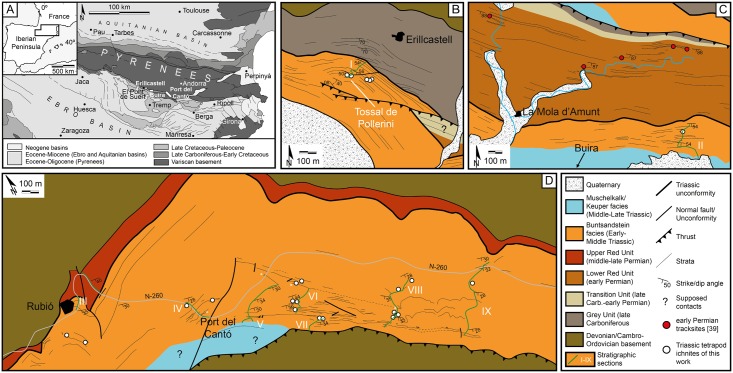
Regional setting and geological maps. (A) Situation of the localities in the Pyrenees. (B-D) Detailed geological maps of Erillcastell (B), Buira (C) and Port del Cantó (D) localities.

In this contribution we present and discuss a diverse Triassic tetrapod ichnoassociation from the Catalan Pyrenees (NE Iberian Peninsula; Fig 1) framed in its stratigraphic and sedimentological setting. The reconstructed (ichno-) faunal assemblage and paleoenvironment (an archosauromorph-dominated fluvial setting) shed light on the paleobiogeography of the Early—Middle Triassic transition, as well as on the onset of continental vertebrate recovery after the end-Permian mass extinction.

## Geological setting

The Catalan Pyrenees (NE Iberian Peninsula, S Europe) are a significant target to understand the late Paleozoic—early Mesozoic evolution in terms of paleontology and paleobiogeography, as long and continuous sedimentary successions ranging from the late Carboniferous to the Middle Triassic are recorded (Fig 1). The reference studies by Mey et al. [23] and Nagtegaal [24] defined the “post-hercynian” units containing the studied fossils. Séguret [25] and Zwart [26] performed the basic regional geology and mapping, while more recent works focused on tectonics and basin architecture and evolution [27–30]. Fortuny et al. [31] revised the vertebrate paleontological content, and Mujal et al. [8] revised and provided new data on the Permian-Triassic transition.

**Fig 2:**
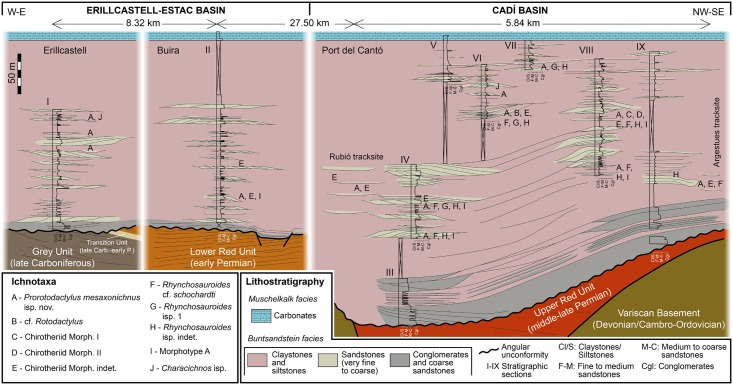
Stratigraphic correlation panel of the three studied localities. Triassic logged sections (I-IX) and location of tetrapod ichnotaxa (A-J).

**Fig 3:**
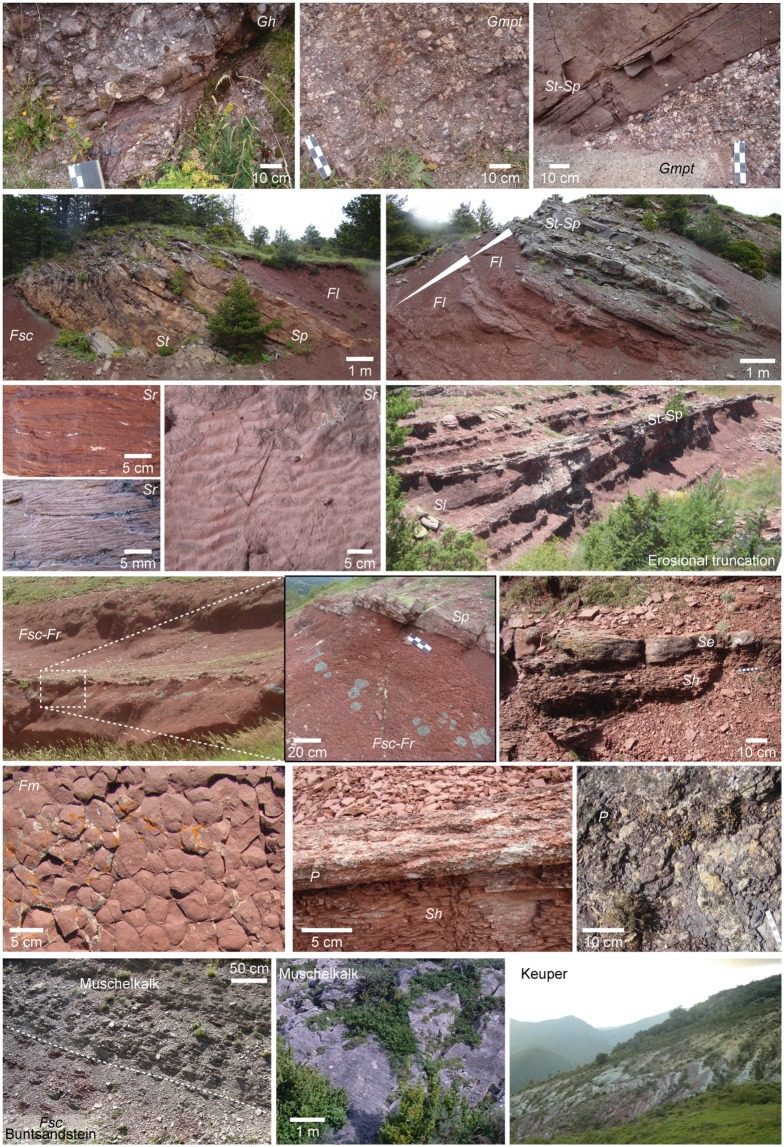
Triassic facies examples from the studied localities of the Catalan Pyrenees. Facies codes correspond to those of the text.

During the earliest Triassic, the continental Pyrenean Basin was arranged in several depocenters resulting from strike-slip (transtensional) tectonics developed in the latest phases of the Variscan cycle [27–30] related with the breakup of Pangea [32]. In this extensional regime, fluviatile sediments covered the Variscan basement and the Carboniferous-Permian volcanosedimentary sequences by means of an erosive angular unconformity (Figs 1 and 2). The resulting deposits are attributed to the syn-rift Buntsandstein facies, later overlaid by post-rift Muschelkalk and Keuper facies (Figs 2 and 3).

## Material and methods

**Fig 4:**
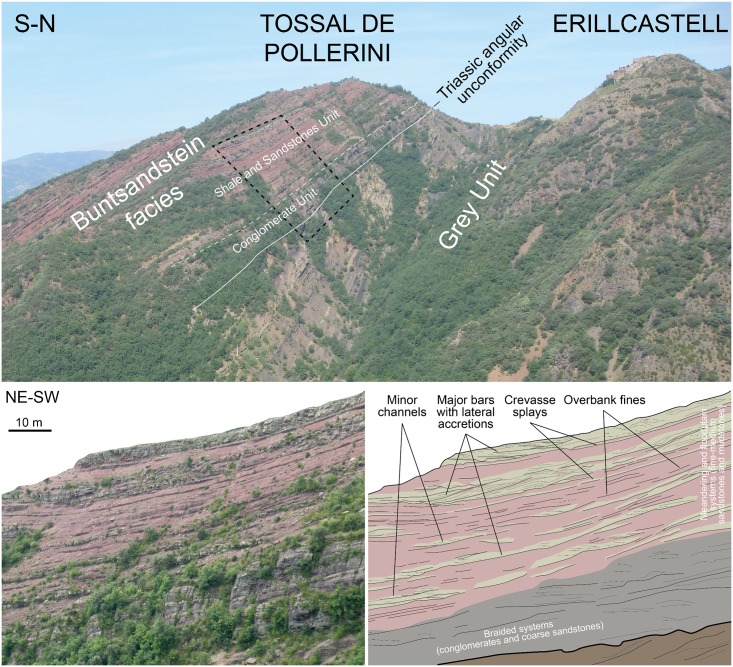
Buntsandstein fluvial facies at the Erillcastell locality, including the Tossal de Pollerini tracksite. The dashed black square in the top picture corresponds to the detail of the fluvial systems in the bottom photo.

In order to provide a detailed geographical and geological framework, we mapped three fossil-bearing localities based on field tracking of strata and photointerpretation (Fig 1). We logged (by means of a Jacobs staff and a centimeter) and correlated bed-by-bed a total of nine stratigraphic sections (numbered I to IX from West to East; Fig 2). The sedimentary facies and architectural elements (Figs 3 and 4) were classified following nomenclature of Miall [33, 34] and further compared with those of Gretter et al. [30].

Tetrapod tracks and trackways (some represented in plastic films) were measured on both specimens and photographs (using ImageJ v.148, http://rsbweb.nih.gov/ij/) following Haubold [35, 36], Leonardi [37] and Ptaszyński [13]. Ichnites were outlined in transparency films and finally digitized with a vector-based drawing software. 3D photogrammetric models of selected specimens were performed using photographs obtained with a digital compact camera Sony T-200 8.1 megapixel following the procedures of Mallison and Wings [38] and processed with three open access software following the procedures of Mujal et al. [8, 39]: Visual SFM (v.0.5.22, http://www.ccwu.me/vsfm/) to generate the point cloud, MeshLab (v.1.3.2, http://meshlab.sourceforge.net/) to perform and edit the mesh (texture, scale, orientation) and ParaView (v.4.1.0 http://www.paraview.org) to generate a color depth map and contour lines. High-resolution molds of tracks and trackways that could not be recovered were made with a two-component polyaddition silicone Harduplex^®^ 23SH, acrylic resin (Acrystal Prima^®^) and glass-fiber. Once in the laboratory, a cast of each mold was produced with the same acryclic resin. Most of the studied specimens were found *in situ* and remained in the field, but those recovered and molds and casts are stored at the Institut Català de Paleontologia Miquel Crusafont.

### Abbreviations

Institutional abbreviation: IPS, Institut Català de Paleontologia Miquel Crusafont, Sabadell, Spain. Studied specimens: IPS-73754, IPS-82611, IPS-82612, IPS-82613, IPS-82614, IPS-82615, IPS-82616, IPS-82617, IPS-82618, IPS-82619, IPS-82619, IPS-82620, IPS-82621, IPS-82622, IPS-82623, IPS-83739, IPS-83740, IPS-83743, IPS-83747, IPS-83748, IPS-83749, IPS-83750, IPS-83753, IPS-88716, IPS-88717, IPS-93867, IPS-93868, IPS-93869, IPS-93870, IPS-93872, IPS-93873, IPS-93874, IPS-93875

Sedimentary facies and architectural elements codes are modified from Miall [33, 34] and Gretter et al. [30]. Conglomerate facies: *Gh*, *Gmpt*. Sandstone facies: *St*, *Sp*, *Sr*, *Sh*, *Sl*, *Se*. Very fine-grained sandstone and mudstone (siltstone and claystone) facies: *Fl*, *Fsc*, *Fm*, *Fr*. Paleosol facies: *P*. Architectural elements: *CH1* (coarse-grained channels), *CH2* (medium- to fine-grained channels), *GB* (gravel bars), *SB* (sandy bedforms), *LA* (sand lateral accretions), *LS* (laminated sand sheets), *OF* (overbank fines).

### Nomenclatural acts

The electronic edition of this article conforms to the requirements of the amended International Code of Zoological Nomenclature, and hence the new names contained herein are available under that Code from the electronic edition of this article. This published work and the nomenclatural acts it contains have been registered in ZooBank, the online registration system for the ICZN. The ZooBank LSIDs (Life Science Identifiers) can be resolved and the associated information viewed through any standard web browser by appending the LSID to the prefix "http://zoobank.org/". The LSID for this publication is: urn:lsid:zoobank.org:pub:B6C36C28-68EF-4F02-B6DD-3A0C8760CCD2. The electronic edition of this work was published in a journal with an ISSN, and has been archived and is available from the following digital repositories: PubMed Central, LOCKSS.

## Stratigraphy and sedimentology

The fossil-bearing unit corresponds to the redbed Buntsandstein facies, analyzed in three different areas, from W to E: Erillcastell (section I, of >151 m thick, including the Tossal de Pollerini tracksite, Erillcastell-Estac basin), Buira (section II, of >226 m, Erillcastell-Estac basin, at 8.32 km from Erillcastell) and Port del Cantó (sections III-IX, of 240 to 350 m, including the Rubió and Argestues tracksites, Cadí basin, at 27.52 km from Buira) (Figs 1 and 2).

Three main units (*sensu* [28]) can be distinguished within the Buntsandstein sequences, from base to top: (a) conglomerate unit (oligomictic conglomerates of quartz pebbles and coarse sandstone with occasional breccia levels with lydite fragments), (b) shale and sandstones unit (reddish and greenish to greyish medium-coarse sandstones with some discontinuous conglomerate levels, reddish very fine to fine sandstones and reddish shales), and (c) shale unit (reddish shales with occasional thin but continuous sandstone beds as those of the shale and sandstones unit). It is worth noting that the thickness of each unit varies according to the basin configuration, and in some cases the conglomerate unit is even missing.

We identified 13 lithofacies (Fig 3), grouped in seven facies associations and constituting architectural elements characteristic from channel to floodplain environments (Fig 4). The sedimentological interpretations are based on our field observations and follow those of Miall [33, 34] and Gretter et al. [30]. Two facies (*Gh* and *Gmpt*) are conglomeratic (commonly of quartz pebbles, and either massive or with cross stratification and other water flow structures), mainly located and well developed in the basal conglomerate unit and constituting deposits of braided systems, such as channels and longitudinal bars (architectural element *CH1*) and gravel bars (architectural element *GB*). Six sandstone facies are found (*St*, *Sp*, *Sr*, *Sh*, *Sl* and *Se*), with grain sizes ranging from very coarse to very fine, and a wide variety of internal stratifications and water flow structures. In the conglomerate unit, facies *St* and *Sp* are in association with facies *Gh* and *Gmpt*, constituting the architectural element *CH1*, and all the sandstone facies constitute channel fills and minor bars of braided systems (architectural element *SB*). In the shale and sandstones unit, and occasionally in the shale unit, sandstone facies occur as meandering systems elements, constituting sandy bedforms (e.g., crevasse splay deposits) associated to lateral accretions (architectural elements *SB* and *LA*, respectively). Facies *St* and *Sp* are also displayed as small channels (in association with facies *Sr* and occasionally *Gh*; architectural element *CH2*). The laminated sand sheets (architectural element *LS*), such as crevasse splays, of the shale and sandstones unit are mainly constituted by facies *Sh* and *Sl*. Scour fill deposits are mostly constituted by facies *Se*. Four facies are related to siltstones and claystones with occasional very fine sandstones (*Fl*, *Fsc*, *Fm* and *Fr*). They mainly constitute floodplain deposits (with common greenish reduction marks associated to plant roots) associated to the meandering systems, which occasionally underwent subaerial exposure (identified by the desiccation structures). These facies are commonly displayed as overbank fines (architectural element OF). In the shale and sandstones unit and in the shale unit, levels related to pedogenic processes (paleosols, facies *P*) were identified: (1) scarce thin levels of carbonate nodules and (2) hardened intervals of facies *Fsc* with hydromorphic marks (large green mottles), root traces and slickensides.

A relevant association is the transition to the Muschelkalk facies. The facies *Fsc* of the shale unit are in transition to marls and fine laminated limestones corresponding to transitional/marine facies. At Port del Cantó, a thick sequence of dark red facies *Fsc* evolves to a laminated grey-greenish limestone/marl sequence. Although the top of the Buntsandstein sequence appears covered at Buira, the base of the Muschelkalk crops out and displays a breccia with Buntsandstein facies clasts embedded in a micritic limestone matrix.

The detailed descriptions and interpretations of facies and architectural elements are provided in the supplemental information (S1 Text). In general terms, the Buntsandstein fluvial succession is displayed as a fining upwards sequence grading from coarse-grained braided to finer meandering and floodplain fluvial systems (high to low energy) and is arranged as a growth strata onlapping onto the Carboniferous-Permian sequences or the Variscan basement (Fig 2). The long laterally-extended meandering sand bodies are interbedded within floodplain mudstone intervals.

Concerning the age of the Buntsandstein facies, Calvet et al. [40] provided palynological data from the shale unit, attributed to the early Anisian (early Middle Triassic). More recently, Mujal et al. [8] provided new palynological and ichnological data, giving an age of late Olenekian (late Early Triassic) for the lower-medium part of the shale and sandstones unit.

## Systematic paleontology

**Fig 5:**
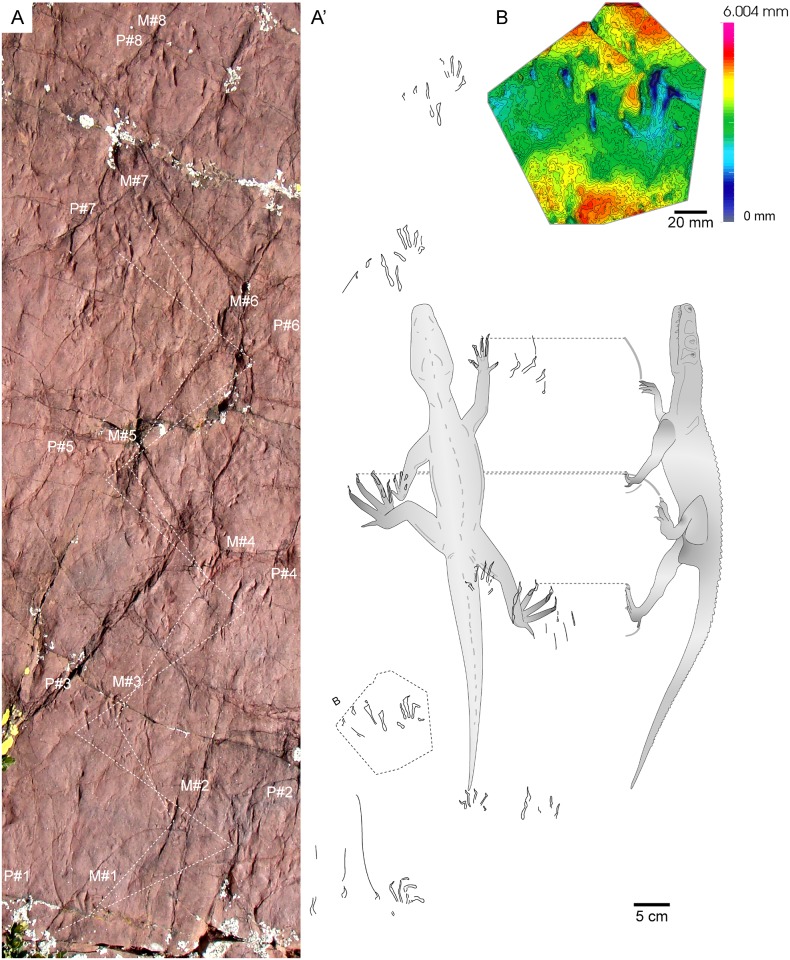
*Prorotodactylus mesaxonichnus* isp. nov. tracks I. (A) Trackway of the holotype (IPS-93870) and outline of the ichnites with the trackmaker (euparkeriid or basal archosauriform) silhouettes in plant view (idealized position in four consecutive tracks) and profile view (position during locomotion). (B) 3D photogrammetric model of a manus-pes set squared in (A).

**Fig 6:**
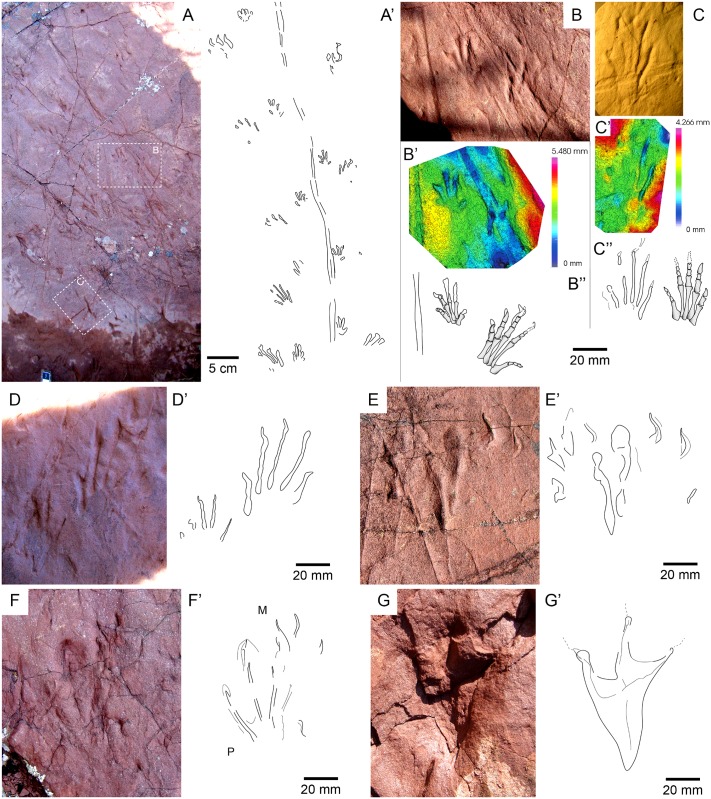
*Prorotodactylus mesaxonichnus* isp. nov. tracks II. (A) Trackway of the paratype IPS-93867 and outline of the ichnites. (B, C) Detail of a manus-pes set (B) and a pes track (C) of the trackway with the 3D models and correlated with the limbs of *Euparkeria capensis* (modified from Nesbitt, 2011 and Bernardi et al., 2015); note that bones and ichnites are at the same scale. (D) Paratype of a well-preserved pes completely overstepping the manus (IPS-93867). (E) Paratype of the manus-pes set of IPS-93871. (F, G) Right pes tracks with prevalence impression of digits II, III and IV (tridactyl function); M and P in (E) refer to manus and pes tracks, respectively.

**Fig 7:**
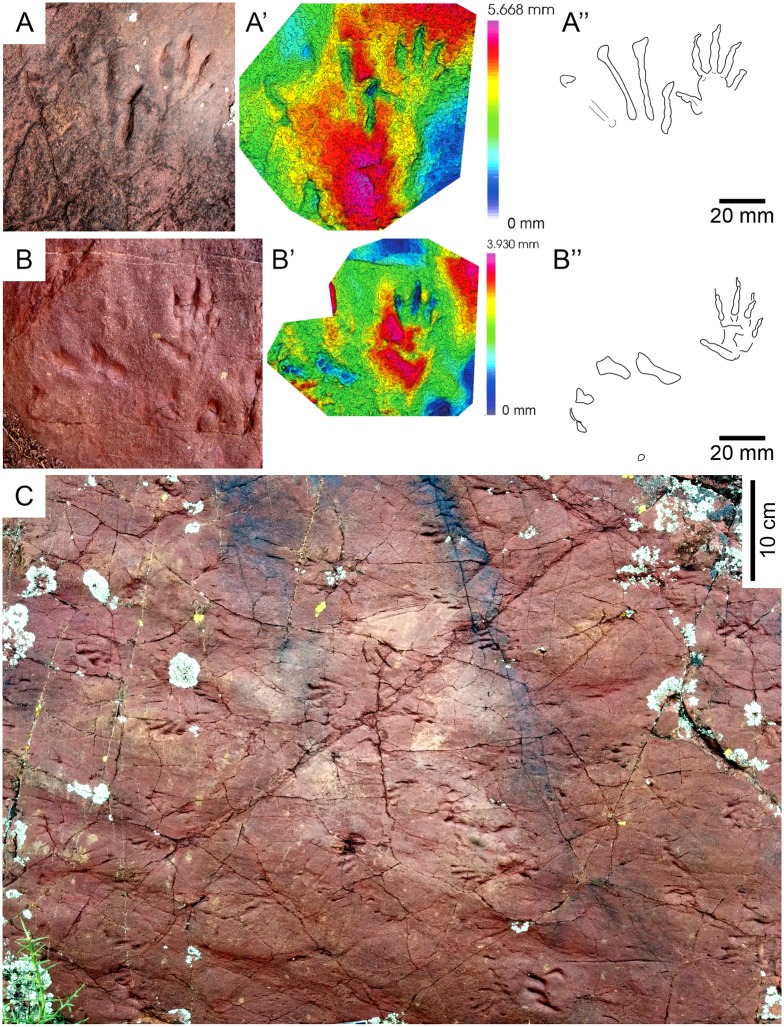
*Prorotodactylus mesaxonichnus* isp. nov. tracks III. (A, B) Manus-pes sets with 3D models and ichnites outline of the paratypes IPS-93868 (A) and IPS-93869 (B). (C) Trampled surface with most of the tracks advancing in the same direction (Westwards).

**Fig 8:**
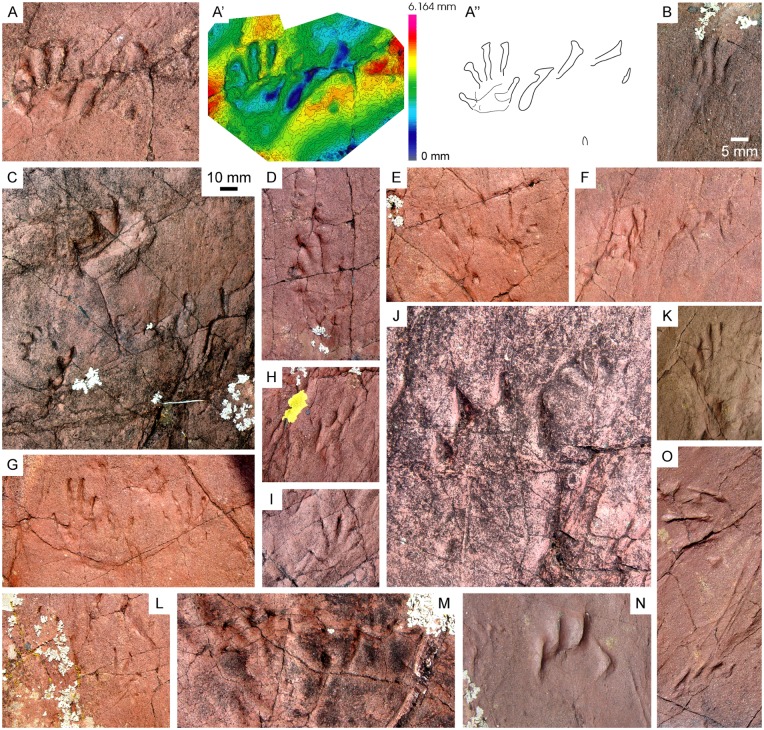
*Prorotodactylus mesaxonichnus* isp. nov. tracks IV. (A) Manus-pes set with the 3D model and ichnites outline. (B-N) Isolated sets, manus and pes tracks with different states of preservation. (O) Pes track with digit scratches and large pes with prevalence of digits II, III and IV. Note the characteristic hooked digit tips (D-G), tracks resembling *Rhynchosauroides* due to the dragging of digit tips (E, I). All tracks scaled at 10 mm except the manus in (B), which is at 5 mm. All tracks are from Tossal de Pollerini site (Erillcastell).

**Fig 9:**
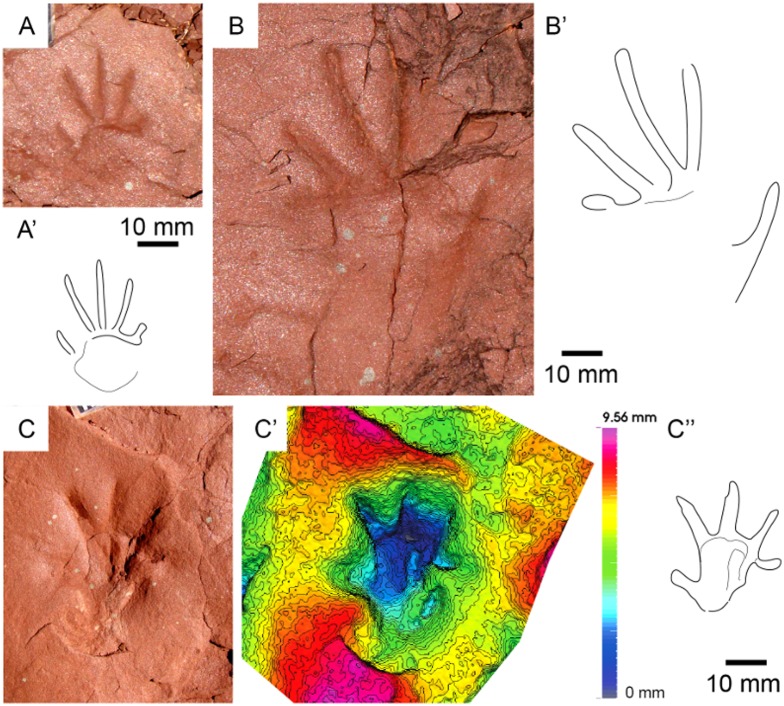
*Prorotodactylus mesaxonichnus* isp. nov. tracks V. Isolated impressions with a different preservation state (probably corresponding to undertracks or overtracks). Note the same kind of preservation despite tracks are small (A, C) or large (B). All tracks are from Port del Cantó, at sections VI (A), VIII (B) and VII (C).

**Fig 10:**
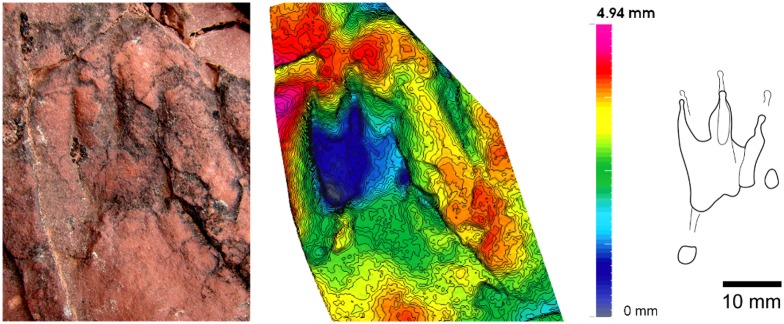
cf. *Rotodactylus* (Port del Cantó, section VI). Isolated manus track with the 3D model and ichnite outline. Note the longest digit III and the backwards rotated digit V.

**Fig 11:**
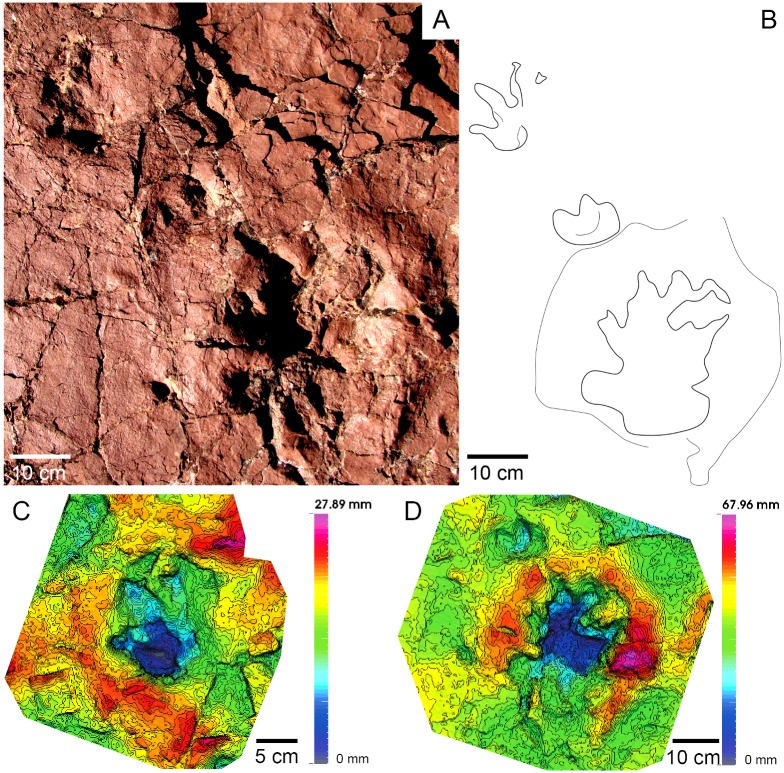
Chirotheriid Morphotype I (Port del Cantó, section VIII). (A) Manus-pes set (mold IPS-82616). (B) Ichnites outline. (C, D) 3D models of manus and pes tracks, respectively.

**Fig 12:**
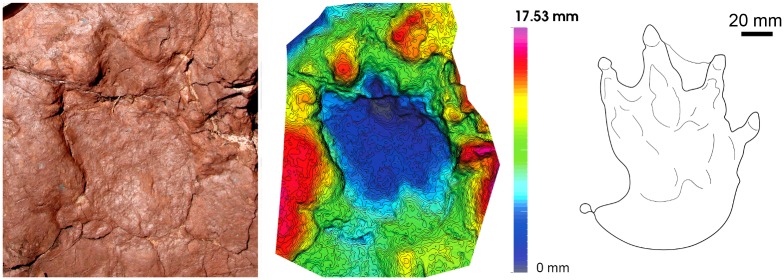
Chirotheriid Morphotype II (Port del Cantó, section VIII). Isolated ichnite with the 3D model and outline.

**Fig 13:**
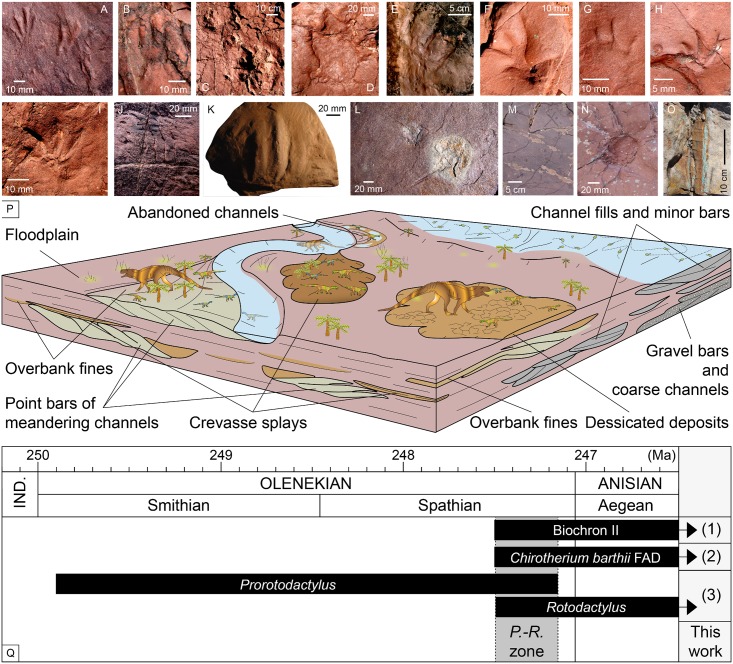
Fossil assemblage, paleoenvironment and biochronologic interval. (A) *Prorotodactylus mesaxonichnus* isp. nov. (B) cf. *Rotodactylus*. (C-E) Chirotheriid tracks: Morphotype I (C), Morphotype II (D) and Morphotype indet. (E). (F-H) *Rhynchosauroides* tracks: *R*. cf. *schochardti* (F), *R*. isp. indet. 1 (G) and *R*. isp. indet (H). (I) Undetermined Morphotype A. (J, K) Swimming scratches of *Characichnos* isp. of small (J) and large (K) size. (L) Arthropod body impressions. (M) Invertebrate burrows. (N) Trunk mark. (O) Plant remain. (P) Paleoenvironmental reconstruction of the Triassic Pyrenean Basin (faunas and plants are not scaled). (Q) *Prorotodactylus-Rotodactylus* zone (*P*.*-R*. zone; grey interval) correlated with the Triassic biochronological intervals (see text for explanation of 1, 2 and 3). FAD: First Appearance Datum. IND.: Induan.

The studied sequences yield a diverse tetrapod ichnoassemblage (Figs 2 and 5–13), including abundant footprints with extramorphological features (precluding ichnotaxonomic assignation) related to locomotion and substrate conditions. Here we provide a detailed description of the identified tetrapod footprint morphotypes. We erect a new ichnospecies and emend the corresponding ichnogenus (monospecific until now), which was only known from the Central European Germanic Basin so far. Some ichnotaxa are equivalent to those of the Palanca de Noves tracksite (12 km Eastwards from Argestues tracksite) [8], thus here we provide additional remarks.

The fossil assemblage of the studied localities also contains invertebrate trace fossils such as arthropod (limulid) body impressions (Fig 13L) and burrows (both horizontal and vertical; Fig 13M), root traces, trunk marks (Fig 13N) and plant stems (Fig 13O). Such remains deserve further analyses out of the scope of this work.

### Ichnofamily Prorotodactylidae Ptaszyński, 2000

#### Ichnogenus

*Prorotodactylus* Ptaszyński, 2000

#### Type ichnospecies

*Prorotodactylus mirus* Ptaszyński, 2000

#### Emended ichnogenus diagnosis

Trackways with small, lacertoid, pentadactyl pes and manus imprints. Manus overstepped laterally by the pes. Pace angulations of pes varying from 70° (low manus overstepping) to 135° (high manus overstepping). Pes outwardly and manus inwardly rotated with respect to the midline. Digits of both manus and pes are clawed. Digitigrade pes with digits I-III increasing in length, digit IV is either the longest or slightly shorter than digit III, divarication angle between digits II and IV ranges from 10° (when digit IV is the longest) to 30° (when digit III is the longest). The metatarsal-phalangeal axis forming posterior end of digits II-IV is straight to slightly anteriorly convex. Digit I everted. Digit V rarely impressed, if present, in a postero-lateral position relative to digits I-IV. Manus semiplantigrade, of chirotheroid shape, compact and rounded with postero-laterally positioned digit V mostly impressed. Digit III always longest, followed by digits IV, II and I which is shortest.

#### New ichnospecies

*Prorotodactylus mesaxonichnus* isp. nov. urn:lsid:zoobank.org:act:B753A1A5-6946-49A5-9785-BA392F18842D (Figs 5–9 and 13A, Tables 1 and 2, S1 and S2 Tables, S1–S4 Figs).

**Table 1:** Minimum, maximum and mean values of the track parameters from the identified trackways of *Prorotodactylus mesaxonichnus* isp. nov.

**Tracks**	**Length**	**Width**	**Digit I**	**Digit II**	**Digit III**	**Digit IV**	**Digit V**	**Length I-IV**
**Minimum Manus tracks**	13.724	12.153	3.653	4.330	6.199	5.347	4.481	9.579
**Maximum Manus tracks**	50.288	35.813	14.831	23.128	33.219	27.521	18.878	41.791
**Total Mean Manus tracks**	37.167	24.088	13.190	17.425	22.953	21.014	12.447	31.248
**Minimum Pes tracks**	32.507	24.367	12.087	14.327	18.977	18.104	13.609*	25.649
**Maximum Pes tracks**	67.090	46.895	29.980	37.095	43.949	42.295	16.731*	57.781
**Total Mean Pes tracks**	45.453	29.989	18.147	22.988	27.475	25.215	15.170*	38.602
**Tracks**	**Width I-IV**	**Div. I-II**	**Div. II-III**	**Div. III-IV**	**Div. IV-V**	**Div. II-IV**	**Div. I-IV**	**Div. I-V**
**Minimum Manus tracks**	8.321	11.138	5.603	5.584	7.585	11.944	35.359	56.644
**Maximum Manus tracks**	26.957	40.409	23.523	21.858	83.372	40.771	68.518	142.220
**Total Mean Manus tracks**	22.867	20.748	15.731	11.883	33.477	27.494	48.606	84.424
**Minimum Pes tracks**	17.200	7.715	3.701	6.919	15.905	16.103	29.525	45.430
**Maximum Pes tracks**	53.513	19.818	21.135	20.917	57.596	40.805	58.114	87.825
**Total Mean Pes tracks**	29.657	14.348	13.240	12.744	33.768	25.941	40.177	71.865

*The mean length of the pedal digit V is not representative, as it is observed in few tracks and usually only preserved by the tip impression. Units in mm and degrees.

**Table 2:** Mean values of the parameters from each identified trackway of *Prorotodactylus mesaxonichnus* isp. nov.

	IPS-93870	IPS-93867	S1A Fig	S1B Fig	S1C Fig	S1D Fig
**Stride manus**	294.884	136.039	277.403	250.021	374.792	-
**Stride pes**	286.771	138.725	267.456	250.335	-	-
**Pace manus**	182.603	89.830	156.814	144.288	202.42	72.23
**Pace pes**	257.069	156.955	204.186	228.978	-	82.615
**Pace angulation manus**	111.485	95.018	119.996	118.285	135.020	-
**Pace angulation pes**	69.668	53.437	78.700	66.246	-	-
**Width pace manus**	102.727	60.854	78.889	73.074	75.688	-
**Width pace pes**	211.174	138.184	160.036	189.776	-	-
**Manus-Pes distance**	62.802	49.778	46.597	59.786	49.782	32.072
**Div. manus midline**	7.074	-3.601	3.084	0.629	-6.629	0
**Div. pes midline**	51.073	46.983	30.608	49.283	72.552	-
**Div. manus-pes digits III**	38.361	42.867	29.567	48.497	92.439	-
**Glenoacetabular distance**	178.161	96.440	145.866	141.065	-	-
**Glenoacetabular Standard deviation**	21.537	5.581	10.014	5.563	-	-

Units in mm and degrees.

#### Etymology

*mesaxonichnus* refers to the longest digit III (mesaxony) of both manus and pes tracks.

#### Holotype

Trackway composed of 8 manus-pes sets (mold and cast IPS-93870; Fig 5).

#### Paratypes

Trackway with tail trace (mold and cast IPS-93867; Fig 6A and 6B), manus-pes sets settled in different trackways (molds and casts IPS-93867, IPS-93868 and IPS-93869; Figs 6D, 7A and 7B, respectively), isolated tracks and manus-pes sets corresponding to IPS-88716, IPS-88717, IPS-93872, IPS-93873 and IPS-93875.

#### Locality and horizon

Tossal de Pollerini tracksite (at 143.8 m of section I, Buntsandstein facies of Erillcastell, Pyrenean Basin), late Olenekian (Early Triassic). El Pont de Suert (Alta Ribagorça, Catalonia, Spain). GPS coordinates (WGS 84 UTM 31T): 318791E, 4698889N.

#### Referred specimens and stratigraphic position

At Erillcastell (Tossal de Pollerini tracksite), several trampled surfaces with five recognized trackways (including those of the holotype and paratypes), manus-pes sets and isolated tracks (>200 tracks) at 96.00 m, 115.00 m and 137.00–143.90 m of section I, and one *ex situ* isolated track (IPS-93874). At Buira, one trackway and several manus-pes sets and isolated tracks at 31.00 m of section II. At Port del Cantó, several isolated ichnites and manus-pes sets (including IPS-82613, IPS-82617, IPS-82618, IPS-82623, IPS-83749), one poorly preserved trackway (mold IPS-82619) at 72.10 m of section VIII, and isolated tracks at 0–0.60 m and 41.30 m of section IV, at 2.00 m and 30.70 m of section VI, at 1.30 m and 2.40 m of section VII, at 0.70–1.20 m, 68.00 m (including mold IPS-82614 from a deformed ichnite), at 70.50 m and 72.10 m of section VIII, and at tracksites of Rubió (including IPS-83753) and Argestues (including IPS-83739 and IPS-83740).

#### Diagnosis

*Prorotodactylys mesaxonichnus* isp. nov. is distinguished from other specimens of the ichnogenus by the following combination of character-states: pes tracks digitigrade (digits I, II, III) to unguligrade (digit IV) with digit V rarely impressed and with a relative digits length of I<V<II<IV<III. Pedal digit impressions are straight to slightly curved and separated one of each other, with digit I slightly posteriorly positioned respect to digits II, III and IV and digit V rotated outwards. The metatarsal-phalangeal axis forming posterior end of digits II-IV is always slightly anteriorly convex. Mean pace angulation is about 118° in manus and 70° in pes tracks. A slightly sinuous tail trace is preserved in some trackways.

#### Description

*Tracks*—Pentadactyl longer than wide ichnites. Manus tracks (14–41 mm long) are usually semiplantigrade but also digitigrade, pes tracks (38–60 mm long) are digitigrade to unguligrade (Figs 5–8 and 13A, S1–S4 Figs, Table 1, S1 Table). The shape of the manus tracks is similar to that of chirotheriid pes footprints. Manus and pes footprints are similar in digit proportions, positions and angulations. In both, digit III is the longest, followed by digit IV and digit II. Digit I is much shorter than the other digits. The length of digit V is between digits I and II, and in some manus tracks digit V presents a relatively large pad impression, starting below digit IV. Digits I to IV are straight, digit V is also straight, but posteriorly positioned and directed outwards. Some manus tracks present scratches connecting to the digit tips, which became slightly curved inwards, and digit IV presents a slightly longer trailing scratch than digit III. Manus are usually deeper and commonly better preserved than pes tracks. In manus tracks, digits II, III and IV are the most deeply impressed (digits deepness: III>IV≥II) and form a compact group with a slightly concave proximal line (i.e., the proximal margin of digit III is in a slightly more anterior position that those of digits II and IV; Figs 5B, 6B, 7A, 7B and 8A). In the manus tracks, digit I is close to digits II, III and IV, but posteriorly positioned. A large expulsion rim is observed between manus digits IV and V (Figs 5B, 7B and 8A). In pes tracks, digit I is posterior to digits II, III and IV (forming a slightly convex arch) as in manus tracks. The pedal digits I and II are “bottle-shaped” (the proximal part with an elongated oval-shape, the medial-distal part being the narrowest and the distal part and the tip nearly as wide as the proximal part). The deepness of digits decreases from I to V in pes impressions, digits I and II being much deeper than the rest. Pedal digit IV is commonly preserved by the tip and a faint impression of the phalangeal portion (Figs 5B, 6B–6E, 7A, 7B and 8A). Pedal digit V is preserved in few tracks and mostly by the tip (Figs 6B, 6E, 6F, 7B and 8A), the phalangeal impression is rarely observed (Fig 6C and 6D), showing that digit V is slightly longer than digit I. Divarication of digits I-IV is relatively low (48.4° in manus and 40.7° in pes tracks; Table 1, S1 Table). Phalangeal pads are well-preserved in some manus tracks (formula 2-2-3-2-2; Fig 7A and 7B, S2 Fig). Some pes tracks also preserve faintly impressed phalangeal pads (Figs 6B, 6C and 7A). The tips of digits I to IV in both manus and pes tracks are often characteristically hooked (convex to the inner part), with an asymmetric T-shape. Some pes tracks present a particular shape, mostly preserving digits II, III and IV (being digit III the deepest), which are divergent and giving a symmetric shape to the impressions (Figs 6F, 6G and 8O). These pes footprints are mostly isolated or in manus-pes sets (but not included in trackways), but are found in surfaces with tracks with the previous described shape and with digit tip scratches. In the area of Port del Cantó several isolated tracks with the morphological traits described above can be identified (Fig 9, S3 and S4 Figs), although the footprints are smoothed as they probably correspond to undertracks or overtracks, and/or were possibly deformed or partially eroded by the water flow soon after their impression.

*Trackways*—Six trackways of alternating manus-pes sets are recognized (Figs 5A and 6A, S1 Fig). The pace angulation of the manus varies from 94.5° to 135° and is commonly about 118°. The pace angulation of the pes varies from 59° to 90° and is commonly of 64–74° (Table 2, S2 Table). Pes tracks are commonly postero-laterally (but also laterally) positioned respect to manus, and rotated outwards (commonly 45–55°) from the midline. When settled in sets, the pes tracks with symmetric shape (prevalence of digits II, III and IV) completely overstep manus tracks (pes tracks are antero-laterally positioned with respect to the manus). Manus tracks are mostly parallel to the midline, but in some specimens are rotated either inwards or outwards (Figs 5A and 6A, S1 Fig). Some pes tracks have arched (convex outwards) digit scratches (Figs 5A and 8O). Relatively wide and slightly sinuous tail impressions are preserved in some trackways, as in the paratype IPS-93867 (Fig 6A, S1A and S1C Fig).

#### Discussion

The relative length of the manus digits (I<V<II<IV<III) and the general shape of the manus, being similar to the chirotheriid pes tracks, are diagnostic of *Prorotodactylus* [13, 16, 19]. Nevertheless, several features substantially differ from the unique well-established ichnospecies, *Prorotodactylus mirus*, as well as from the resembling ichnogenera *Rotodactylus* and *Rhynchosauroides*: (a) The pedal digit proportions (with digit III the longest) and their relative deepness (decreasing from I to V), (b) the pes tracks much larger than manus, (c) the pedal digit impressions separated on from each other, (d) the characteristically hooked (convex to the inner part) or also T-shaped digit tips (clawed) of both manus and pedes, (e) the low pace angulations (about 118° in manus and 64–74° in pedes), (f) the postero-lateral position of the pes respect to the manus tracks, (g) the outwardly rotated pedes and the parallel manus from the midline, and (h) the presence of slightly sinuous tail impressions. The trackway parameters (relatively low pace angulations) are similar to those of *Rhynchosauroides*. However, a diagnostic feature of *Rhynchosauroides* is the longest digit IV in both manus and pes tracks, whereas in the Pyrenean tracks digit III is the longest, thus precluding the assignation to *Rhynchosauroides*. Similarly, the chirotheriid pes-like shape of manus tracks is a character shared by both *Prorotodactylus* and the herein reported tracks, but not found in *Rhynchosauroides*. Trackway parameters are linked to the body plan and relative length of trackmakers limbs, but are also highly influenced by the locomotion gait, as well as by the substrate conditions. Thus, the morphological characters of the tracks (e.g., length, width, relative length of the digits, etc.) present less variation than trackway parameters. In all, the combination of features of these Pyrenean tracks is consistent with the existence of a different and new morphotype for *Prorotodactylus*.

Until now, *Prorotodactylus* tracks were only known from the Germanic Basin [13, 15–19], and possibly from the French Lodève Basin [13, 41]. Ptaszyński [13] reassigned *“Rhynchosauroides lutevensis”* of Demathieu [41] to *Prorotodactylus lutevensis* based on the similarity of the manus tracks with the Polish *Prorotodactylus*. “*Rhynchosauroides lutevensis”* was erected upon a single trackway composed of three manus-pes sets. The characters of the French tracks are also similar to those herein reported (e.g., shape of pedal digits and pes pace angulation of 96°), but differ in the pedal digit IV, which is the longest in the French tracks, as in the Polish *Prorotodactylus*. We agree with Ptaszyński [13] on the assignation of the French trackway to *Prorotodactylus*, but with an open ichnospecies nomenclature, because further and more complete specimens and trackways are needed (e.g., no impressions of the pedal digit V are recognized) [41] and so other features (e.g., pace angulation, relative manus-pes position and relative digits length) are possibly linked to extramorphologies (i.e., substrate conditions and speed locomotion). In the nearby Palanca de Noves site (12 km eastwards from Argestues tracksite), Mujal et al. [8] identified an isolated manus-pes set referred to a *Prorotodactylus-Rotodactylus* plexus. The new Pyrenean tracks described here confirm the presence of *Prorotodactylus* in the Western Tethys, which present differences with the specimens from the Central European Germanic Basin.

The slightly outwardly rotation of some manus, the high expulsion rim in the outer side and the hooked digit tips indicate that the forelimbs were directed outwards during the impression and inwards during the rising, describing a sinusoidal movement. The scratches in some pedal digits describe a laterally-arched movement of the hindlimbs, being similar to that of the elongated trailing traces of the manus digit IV. The wide standard deviation of the glenoacetabular distances (Table 2, S2 Table) and the sinusoidal shape of the tail impression suggest a lateral torsion of the trackmaker body trunk. Together with the low pace angulations and the relative wide trackways, a quadrupedal sprawling to semi-erect locomotion is inferred (Fig 5). The tail impressions, mostly associated to trackways with low pace angulations, would indicate a relatively slow locomotion. Noteworthy, the pedal impressions with a prevalence function of digits II, III and IV (tridactyl shape similar to dinosaur tracks), anteriorly positioned to manus impressions, or not being associated with manus (nor other tracks) suggest a potential occasional faster (i.e., cursorial) locomotion with a tridactyl function of hindlimbs, differing from that of the recognized trackways.

#### The potential trackmakers

In order to correlate the footprints of *Prorotodactylus mesaxonichnus* isp. nov. with their potential trackmaker, a synapomorphy-based approach is carried out by following the methods of Carrano and Wilson [42]. First, these footprints must be compared with other ichnological records. The most similar known ichnotaxa are the Triassic *Prorotodactylus*, *Rotodactylus* and *Rhynchosauroides*. The chirotheriid pes-like shape of the manus is diagnostic of *Prorotodactylus*, whereas the trackway parameters (low pace angulations, relative manus-pes distance) resemble those of *Rhynchosauroides*. Therefore, the trackmaker most probably presented morphological similarities (by phylogenetic relation and/or morphological convergence) to the trackmakers of these ichnogenera. The claw marks exclude amphibians, and point to a diapsid trackmaker instead.

Nevertheless, the less inclusive feature is mesaxony (i.e., digit III the longest) in both manus and pes tracks. Regarding the Triassic amniote tetrapod groups, mesaxony in both fore- and hindlimbs is most commonly found in archosauromorphs and derived groups [43, 44]. Brusatte et al. [15] provided a comprehensive review of characters of the Triassic terrestrial tetrapods in their supplementary material. Based on their work, the mesaxony character excludes the Triassic groups of rhynchosaurs, sphenodontians, drepanosaurids, kuehneosaurids, parareptiles and basal diapsids. Other potential trackmakers would be the dicynodont and cynodont synapsids, but their limbs are relatively shorter and thicker (i.e., more robust or stubbier) than their potential impressions. Similarly, the relatively short hindlimbs of dicynodonts and cynodonts would not overstep the forelimbs, and their tails would not be impressed during locomotion. Brusatte et al. [15] concluded that the Polish *Prorotodactylus*, as well as *Rotodactylus*, were probably impressed by dinosauromorphs [19] (but see [16, 17, 45, 46] for an alternative discussion). On the contrary, the quadrupedal trackways of *Prorotodactylus mesaxonichnus* isp. nov. present low pace angulations (i.e., a sprawling or semi-erect posture of the trackmaker). This feature excludes archosaurs as trackmakers, as the narrow gauge trackway is a synapomorphy of the group [15].

Consequently, the most reliable trackmakers of *Prorotodactylus mesaxonichnus* isp. nov. are non-archosaurian archosauromorphs with mesaxonic limbs. Based on the detailed archosaurmorph analyses by Nesbitt [43], in *Euparkeria* both metacarpal and metatarsal III are the longest among them, denoting mesaxonic limbs. In a morphological comparison, the manus-pes proportions and the pedal digits relative length are the same for both *P*. *mesaxonichnus* isp. nov. and *Euparkeria*, and thus equivalent points can be identified in both ichnites and bones (Figs 5A, 6B and 6C). Noteworthy, *Euparkeria* presented a sprawling to semi-erect posture of the humerus during locomotion, and the femur was held in a horizontal position during slow locomotion, being able to reach a semi-erect gait in faster locomotion [47, 48]. The glenoacetabular distance of *Euparkeria* [48] correlates with those of the trackways of *P*. *mesaxonichnus* isp. nov. (between 90 to 150 mm; Table 2, S2 Table). The relatively large head of *Euparkeria* also correlates with the better and relatively deeper preservation of manus than pes tracks in *P*. *mesaxonichnus* isp. nov. Sookias and Butler [48] also suggested a potential facultative bipedalism, which may fit with the pedal tracks with prevalence function of digits II, III and IV, linked to occasional cursorial locomotion (Figs 6D, 6F, 6G, 8N and 8O). Therefore, given all these evidences, we suggest euparkeriids (*sensu* [49]) and similar basal archosauriforms as potential trackmakers of *Prorotodactylus mesaxonichnus* isp. nov.

### Ichnofamily Rotodactylidae Peabody, 1948

#### Ichnogenus

*Rotodactylus* Peabody, 1948

cf. *Rotodactylus* (Figs 10 and 13B).

#### Referred specimens and stratigraphic position

At Port del Cantó, isolated tracks (including mold IPS-82611) at 2.00 m of section VI.

#### Description

Small elongated pentadactyl ichnites. The digits relative length is I<II<IV≤III. Digit V is rotated posteriorly, preserved mostly by the tip. Digit I is reduced, preserved as a rounded tip impression. Digits II, III and IV are much longer than digit I, and present small claw marks. The proximal parts of digits II, III and IV are deeper impressed than the tips. The digits deepness increases from II to IV.

#### Discussion

The longer than wide footprints, the relative digits length, and the position and shape of digit V are diagnostic traits of *Rotodactylus* [16, 19, 50]. The scarcity of footprints and lack of trackways prevent a confident assignation, being preferable its confer to *Rotodactylus*. The slightly longer digit III than digit IV indicates that this footprint may correspond to a manus impression, as in *Rotodactylus* only in some manus tracks the digit III has been identified as the longest [16, 19]. In the Early and Middle Triassic of Poland, Germany, France, Eastern Spain and the USA this ichnogenus is much more abundant [16, 31, 35, 36, 50, 51], being the dominant ichnotaxon in some Middle Triassic localities [19]. The potential trackmakers are still matter of discussion, *Rotodactylus* has been referred to dinosauromorphs [15, 19], but also to non-dinosauromorph archosauromorphs [16] such as basal archosauromorphs [46].

### Ichnofamily Chirotheriidae Abel, 1935

#### Morphotype indet. I

Chirotheriidae Morphotype I (Figs 11 and 13C).

#### Referred specimens and stratigraphic position

At Port del Cantó, one manus-pes set and another associated manus track (mold IPS-82616) at 68.00 m of section VIII.

#### Description

The pes track is pentadactyl and plantigrade to semiplantigrade, of approximately 25 cm long. Digit III is the longest, and digit I the shortest. Digits I to IV are wide, of triangular shape, straight and subparallel, with clawed tips. Digit V is in a posterior position, curved outwards, and preserving a large pad impression. The two small tracks correspond to manus impressions. The closest manus to the pes is semiplantigrade, and preserves three digits, most probably digits II, III (the longest) and IV. The other manus track is semiplantigrade and of approximately 10 cm long, with four digits preserved. The shape is similar to that of the pes track, but with slender digits and with an angle between the digits II, III and IV higher than in those of the pes track.

#### Discussion

The shape of the tracks, and the manus-pes proportions are features diagnostic of the chirotheriid ichnofamily [35]. The lack of trackways and the poor footprint preservation preclude a confident assignation, but regarding the late Early—early Middle Triassic ichnoassemblages [9, 14, 16, 52, 53], the pedal footprint outline, the robustness of digits I to IV and the large pad of digit V, these tracks show some affinities to *Protochirotherium*. The potential trackmakers of such tracks may correspond to basal archosauriforms, such as erythrosuchids or crocodilian-stem archosaurs [53].

#### Morphotype indet. II

Chirotheriidae Morphotype II (Figs 12 and 13D).

#### Referred specimens and stratigraphic position

At Port del Cantó, one isolated track in concave epirelief at 68.00 m of section VIII.

#### Description

Pentadactyl, semiplantigrade-digitigrade, and longer than wide track (about 13 cm long). Digit III is the longest, followed by digit IV and digit II. Digit I is the shortest, being 1/3 in length of digit III. Digits I to IV form a group, separated from digit V. Digit V presents a wide pad, and is outwardly oriented. The angulation of digits I-II and II-III is higher than that of digits III and IV, which are nearly parallel. Claw marks are observed in all the digit tips. The footprint is deeper in the anterior and central part (the heel is the shallowest part).

#### Discussion

The concave posterior margin is similar to that of archosaur footprints, corresponding to the overlap of the metatarsals II and IV [16, 54]. The reduced and posterior position of digit I, as well as the position of digit V, are characteristic of manus tracks of *Protochirotherium* [53, 54], but as no other tracks are associated, the assignation remains open.

#### Morphotypes indet

Chirotheriidae Morphotypes indet. (Fig 13E, S5 Fig).

#### Referred specimens and stratigraphic position

At Buira, one isolated track in concave epirelief at 31.00 m and 69.00 m of section II. At Port del Cantó, one manus-pes set and about a total of 10 isolated ichnites at tracksites of Rubió and Argestues (including IPS-83739), at 48.00 m of section IV, at 0.60 m of section VI, and at 68.00 m of section VIII (including IPS-83750).

#### Description

The footprint of Buira (Fig 13E) corresponds to a large impression (>12 cm long) with three long digits. The middle digit is the longest. The three preserved digits are nearly parallel, they present triangular shape with clawed tips, and are slightly curved toward middle digit axis (which is straight). The isolated manus-pes set of Port del Cantó corresponds to two rounded impressions (S5A Fig), one much larger than the other (of 8 and 20 cm long), with high expulsion rims similar to those of the chirotheriid Morphotype I. The IPS-83750 of Port del Cantó corresponds to a partial track preserving three smooth digits, probably corresponding to III, IV and V (S5C Fig). The estimated size of the track is equivalent to that of Morphotype I (20–25 cm long).

#### Discussion

These tracks preserve few features to confidently be assigned to any ichnogenus. Nevertheless, the size and digit shapes are that of the chirotheriid ichnofamily [35, 54]. The footprint of Buira resembles those of chirotheriids from the nearby Palanca de Noves tracksite [8].

### Ichnofamily Rhynchosauroidae Haubold, 1966

#### Ichnogenus

*Rhynchosauroides* Maidwell, 1911

#### Ichnospecies—I

*Rhynchosauroides* cf. *schochardti* (Rühle von Lilienstern, 1939) (Fig 13F, S6A–S6C Fig).

#### Referred specimens and stratigraphic position

At Port del Cantó, isolated tracks at 0–0.60 m and 41.30 m of section IV, at 2.00 m of section VI (including molds IPS-82612, IPS-82619), at 0.70–1.20 m, 68.00 m, 70.50 m and 72.10 m of section VIII, and isolated tracks *ex situ* at Argestues tracksite.

#### Remarks

The characteristically asymmetrical footprints, with the long and slender digits I to IV (in increasing length) curved inwards and digit V curved and rotated outwards, the digits angulation, and the relatively sharp, hooked-shape heel are features of *Rhynchosauroides schochardti* manus tracks. This ichnospecies characteristic from the Early—Middle Triassic [11, 20, 35, 36, 55]. These footprints present the same shape as those manus tracks conferred to *R*. *schochardti* at Palanca de Noves. The *Rhynchosauroides* ichnogenus is commonly related to archosauromorph and lepidosauromorph trackmakers [16, 20].

#### Ichnospecies—II

Undetermined *Rhynchosauroides* ichnospecies 1 of [8] (Fig 13G, S6D Fig).

#### Referred specimens and stratigraphic position

At Port del Cantó, several isolated tracks at 41.30 m of section IV, at 2.00 m of section VI, and at 1.30 m of section VII (including molds IPS-82620, IPS-82621, IPS-82622).

#### Remarks

The slightly longer than wide footprints, the deepness of the digits and the outwardly curved digits (especially digit IV) are features of the *Rhynchosauroides* isp. 1 described at Palanca de Noves [8]. The lack of track couples or sets precludes assigning these tracks to manus or pes impressions.

#### Ichnospecies—III

Undetermined *Rhynchosauroides* ichnospecies of [8] (Fig 13H, S6E–S6G Fig).

#### Referred specimens and stratigraphic position

At Port del Cantó, several isolated ichnites at 0–0.60 m and 41.30 m of section IV, at 2.00 m of section VI, at 2.40 m of section VII (including IPS-83747 and IPS-83748), and at 0.70–1.20 m and 68.00 m (including mold IPS-82615) of section VIII.

#### Remarks

These *Rhynchosauroides* footprints are characterized by their small size and the angulation of digits IV-V at approximately 90°. As at Palanca de Noves [8], these footprints could correspond to juvenile specimens of other *Rhynchosauroides* forms. As no sets nor trackways are preserved, impressions cannot be assigned to pes or manus, and ichnospecies cannot be determined.

### Morphotype indet. A

Undetermined Morphotype A of [8] (Fig 13I, S7 Fig).

#### Referred specimens and stratigraphic position

At Buira, isolated ichnites at 31.00 m of section II. At Port del Cantó, isolated ichnites (including IPS-73754) at 0–0.60 m and 41.30 m of section IV, 0.70–1.20 m and 68.00 m of section VIII.

#### Remarks

Ichnites are pentadactyl, with digit IV slightly shorter than digit III (the longest). Digit I is preserved by a shallow tip impression. Digit V is posteriorly positioned from digit group I-IV and slightly rotated outwards. Digits are slightly curved inwards, with clawed tips. Despite some impressions are digitigrade, they are commonly plantigrade to semiplantigrade, with a wide oval-shaped palm with an elevated ridge separating it from digits II, III and IV. These tracks correspond to manus impressions [8]. Digit proportions are those of *Prorotodactylus*, but the wide oval palm is not observed in this ichnogenus. At the analogous Palanca de Noves tracksite, Mujal et al. [8] reported several footprints and a faint trackway attributable to an archosauromorph trackmaker.

### Ichnogenus *Characichnos* Whyte and Romano, 2001

*Characichnos* isp. indet. (Fig 13J and 13K).

#### Referred specimens and stratigraphic position

At Erillcastell, small scratches at 138.20 m, 142 m and 143.90 m of section I. At Port del Cantó, large track (IPS-83743) at 39.50 m of section VI.

#### Description

Two main track sizes are recognized. The traces from Erillcastell correspond to small digit tip scratches, with widths of 2–3 cm and variable lengths (from 4–5 cm to up to 30 cm; Fig 13J). The marks are long and continuous, being straight to sinuous, and in groups of three scratches. IPS-83743 corresponds to a large (7 cm wide and 10 cm long) crocodile-like track composed of four sigmoid-shaped digit scratches (Fig 13K).

#### Discussion

These kinds of scratches are interpreted as swimming traces [21, 22, 56]. The small scratches from Erillcastell may have been impressed during a flooding event, as are associated with other walking-gait ichnites of *Prorotodactylus mesaxonichnus* isp. nov. The size of these small scratches is comparable to the tracks of *P*. *mesaxonichnus* isp. nov., thus they were probably impressed by the same trackmaker. The large track IPS-83743 of Port del Cantó fits with the size of chirotheriid tracks [22]. It was found at the base of a meandering channel (facies *St*), hence impressed under a relatively deep water column considering the size of the track.

## Discussion

### Paleoenvironmental reconstruction of the Triassic Pyrenean fossil record

Stratigraphic and sedimentological data allow to determine the environmental conditions of the studied successions (Fig 13P, Table 3). The upper part of the basal conglomerate unit is constituted by sequences of facies *St-Sp* (Fig 2), where cylindrical burrows (mostly vertical) are common and scarce plant remains are also present, demonstrating the general high energy water flow of the braided system. Some surfaces yield abundant arthropod (limulid) body and trace fossils (Fig 13L), indicating episodes of relatively low water flow energy on the braided systems. Deeper environmental affinities of such arthropods in Early Triassic deposits deserve further analyses out of the scope of this work.

**Table 3:** Facies with tetrapod footprints, plant stems and impressions, and invertebrate ichnites.

**Port del Cantó**		
**Facies associations**	**Architectural elements**	**Fossils**
St-**Sp**	Sandy bedforms (SB)	Limulid body and trail impressions, invertebrate cylindrical burrows, plant stems
**Fl**	Overbank fines and abandoned channels (OF)	*Rhynchosauroides* isp. indet.
Sl-**Fm**	Crevasse splays (SB) and abandoned channels (OF), some surfaces are mud-cracked	*Prorotodactylus mesaxonichnus* isp. nov., all *Rhynchosauroides* morphotypes, plant stems and trunk marks, and abundant burrows (in the uppermost level, with trough cross stratification and flow ripples)
**St** → Sh → St	Point bars (LA) of grey sandstones	Plant stems (with abundant malachite)
Fl-**Fm**	Abandoned channels (OF)	*Prorotodactylus mesaxonichnus* isp. nov., all chirotheriid morphotypes, *Rhynchosauroides* cf. *schochardti*, *Rhynchosauroides* isp. indet., Morphotype A
**Sp**	Crevasse splays (SB)	*Prorotodactylus mesaxonichnus* isp. nov., Morphotype A, Chirotheriid Morph. indet., trunk marks
**Sr** → Fsc	Crevasse splays (SB)	*Prorotodactylus mesaxonichnus* isp. nov., plant stems
**Fl**	Abandoned channels (OF)	*Prorotodactylus mesaxonichnus* isp. nov., cf. *Rotodactylus* isp. Chirotheriid Morph. indet., *Rhynchosauroides* cf. *schochardti*, *R*. isp. indet.
**St** → Sr	Crevasse splays (SB)	*Prorotodactylus mesaxonichnus* isp. nov.
St	Point bars (LA)	Large scratch track of *Characichnos* isp.
Sl	Crevasse splays (SB)	*Prorotodactylus mesaxonichnus* isp. nov., *Rhynchosauroides* isp. indet.
**Sr/Fl** → Fl	Crevasse splays (SB) and overbank fines (OF)	*Prorotodactylus mesaxonichnus* isp. nov., *Rhynchosauroides* cf. *schochardti*, *R*. isp. indet., and Morphotype A
St → Sl → Fsc (interbedded **Sl**)	Crevasse splays (SB)	*Prorotodactylus mesaxonichnus* isp. nov., all *Rhynchosauroides* morphotypes, and Morphotype A
St/**Sr**	Crevasse splays (SB)	Chirotheriid Morph. indet.
**Buira**		
**Facies associations**	**Architectural elements**	**Fossils**
St → **Sp** → Sr → Fsc (Se)	Point bars (LA) and crevasse splays (SB)	*Prorotodactylus mesaxonichnus* isp. nov., Chirotheriid Morph. indet., *Rhynchosauroides* cf. *schochardti*, *R*. isp. indet, Morphotype A, invertebrate burrows
**St** → Fsc	Crevasse splays	Deformed indet. tetrapod tracks
**Erillcastell**		
**Facies associations**	**Architectural elements**	**Fossils**
**Sp** → Fsc	Crevasse splays (SB)	*Prorotodactylus mesaxonichnus* isp. nov., plant stems and trunk marks
Fm	Overbank fines and abandoned channels (OF)	*Prorotodactylus mesaxonichnus* isp. nov.
Sp-Sr → **Se**-Fsc	Crevasse splays (SB)	*Prorotodactylus mesaxonichnus* isp. nov., *Characichnos* isp.
**Sr** → Fl	Crevasse splays (SB)	*Prorotodactylus mesaxonichnus* isp. nov., *Characichnos* isp.
**St** → Fsc (Se)	Crevasse splays (often mud-cracked; SB) and small point bars (LA)	*Prorotodactylus mesaxonichnus* isp. nov., *Characichnos* isp.

Facies (see codes in corresponding text section and in S1 Text) in bold are those bearing fossils. Conglomeratic and massive mudstone facies do not contain fossils. Arrows indicate gradation of facies.

In the floodplain deposits of the shale and sandstones unit and shale unit, root traces are evidenced by greenish reduction marks that at some points define horizons parallel to the stratification, sometimes related to paleosols (Fig 3). This last feature indicate soil stabilization and energy decrease of the depositional system (final phases of the syn-rift). In the upper surfaces of crevasse splay deposits of 10–15 cm thick plant stem impressions and rounded trunk marks are preserved (Fig 13N), sometimes associated to capped root traces, indicating episodic flooding events of the meandering streams. The thick sandstone deposits of Port del Cantó (section IV), interpreted as large point bars of meandering systems, preserve large plant stems (Fig 13O), probably transported in episodic high energetic flow events of the main rivers streams. Invertebrate burrows are common in fluvial bars of the shale and sandstones unit. In deposits associated to occasional and rapid events (i.e., crevasse splays and scour fill deposits) burrows are vertical, whereas in abandoned channels (low energy systems) burrows are horizontal, long and sinuous (Fig 13M).

The tetrapod footprints are found in the shale and sandstones unit (Figs 2, 4 and 13P, Table 3). They are abundantly preserved in abandoned channels (at facies *Fl* and facies association *Sl-Fm*) and even in small point bars (at facies associations *St-Fsc*, *St-Sr*, and *St-Sp*), as well as in crevasse splay deposits (at facies *Sl* and facies associations *Sr-Sl*, *Sp-Fsc*, and *St-Sl-Fsc*) (Table 3). The low number of swimming scratches may indicate that trackmakers passed through the deposits with a low water level. Only the large chirotheriid scratch is found in well-developed point bar facies (*St-Sp*), thus suggesting that relatively large archosauromorphs probably had swimming faculties and possible water-living activity. Some *Prorotodactylus mesaxonichnus* isp. nov. trackways have asymmetric manus-pes distances (i.e., in one side the fore- and hindlimbs of the trackmaker were closer than in the other during locomotion; Figs 5A and 6A, S1 Fig, S2 Table), suggesting surface sloping towards the side of the more spaced tracks.

Several trampled surfaces with hundreds of *Prorotodactylus mesaxonichnus* isp. nov. footprints advancing in the same direction (westwards) occur in Erillcastell (Fig 7C). This also occurs in Buira, although footprints are not so abundant and an isolated chirotheriid track is also preserved. The trampled surfaces may indicate gregarious behavior and increased activity [15]. Similarly, such abundance of footprints, as well as their wide distribution along the different facies (Table 3), may also denote generalist faunas [8] occupying a wide range of ecological niches. In the same way, different states of footprint preservation (i.e., unguligrade, digitigrade, semiplantigrade, plantigrade) are observed (Figs 5–9, S2–S4 Figs), both in different levels and in the same surfaces. Footprints with large dragging component denote soft substrates, while faint digit tip impressions or claw marks were recorded in relatively dry or hard substrates. Some track-bearing surfaces present either flow structures (e.g., facies *St*, *Sr* and *Se*) or mud-cracks (e.g., facies *Fm*). In sum, this would be the result of substrate varying moisture, a recurrent process in small ponds and crevasses with sporadic flooding (Fig 13P).

### Early Triassic biostratigraphy and paleobiogeography

The Triassic Pyrenean tetrapod ichnoassemblage contributes to the knowledge of the general frame and distribution of the ichnofaunal record. Until now, occurrences of *Prorotodactylus*, which are always associated with *Rotodactylus*, were restricted to the Central European Germanic Basin [15–19], and possibly to the French Lodève Basin [13]. This association of ichnogenera indicates a late Olenekian age [19]. Recently, Mujal et al. [8] provided palynological data giving a late Olenekian age for the basal portion of the Pyrenean Buntsandstein facies. Similarly, Calvet et al. [40] dated as early Anisian the shale unit (uppermost Buntsandstein). Together, these works support a latest Olenekian age of the tetrapod ichnoassociation. In this way, the Pyrenean ichnoassemblage points to a Central Pangean distribution of *Prorotodactylus*, although it could be similar to that of *Rotodactylus*. Therefore, we suggest the *Prorotodactylus-Rotodactylus* zone as a new Triassic biochronological interval for, at least, Central Pangea, constituted by deposits with both ichnogenera (Fig 13Q). This zone probably corresponds to the lower parts of the Triassic biochron II [57] (1 in Fig 13Q) and the First Appearance Datum of *Rotodactylus* and *Chirotherium barthii* [52] (2 in Fig 13Q), coinciding with the overlapping interval of *Prorotodactylus* and *Rotodactylus* (3 in Fig 13; see [19]). Other characteristic late Early Triassic ichnotaxa are *Rhynchosauroides* and large chirotheriid tracks, such as *Protochirotherium* [16, 53], being potentially similar ichnotaxa to those of the Triassic Pyrenean assemblage and denoting a Central Pangean homogeneity of ichnofaunas. The trackmakers of all these ichnotaxa most probably correspond to archosauromorphs. This group radiated in the late Permian [1, 43, 58, 59], and several clades evolved along Pangea, probably in the aftermath of the different Permian extinctions [60].

The pedal and trackway pattern differences of the Pyrenean and Polish *Prorotodactylus* indicate that this ichnogenus includes different trackmakers, being in accordance with the late Early Triassic archosauromorph diversification in the aftermath of the end-Permian extinction event. The mesaxonic pedes and the low pace angulations of *Prorotodactylus mesaxonichnus* isp. nov. are possibly correlated to euparkeriids and basal archosauriforms (*sensu* [44, 49]), whereas *P*. *mirus* (ectaxonic pedes and higher pace angulations) has been referred to small dinosauromorphs [13, 15, 19], although such attribution is controversial [16, 17, 45, 46]. The Polish *Prorotodactylus* (and also *Rotodactylus*) tracks were compared with the limbs of the dinosauromorph *Lagerpeton* [15] and a potential *Lagerpeton*-like dinosauromorph [61]. Nevertheless, the relative digits length and proportions of *Lagerpeton* do not match the relative depths of *Prorotodactylus* digit impressions (e.g., digit I would not be impressed) [46]. Interestingly, the similar manus impressions of the Pyrenean and Polish *Prorotodactylus* may be related to the low morphological disparity of archosauriforms suggested by Ezcurra and Butler [62]. In summary, we suggest stem archosauriforms as potential trackmakers of *Prorotodactylus*, such as euparkeriids for *P*. *mesaxonichnus* isp. nov.

### The Triassic vertebrate recovery

In recent years, several works focused on the diversification of archosauromorphs during the Triassic [43, 44, 49, 60, 62]. Early Triassic footprints are much more abundant than body fossils, as is the case of the Western Tethys region [20, 52, 59], hence the ichnological record plays an important role to understand the vertebrate recovery. The new findings represent the earliest evidence of Mesozoic Iberian vertebrates, and one of the few and the most complete record from the Western Tethys.

The correlation between ichnites and sedimentary environments in the Catalan localities (Table 3) shows that archosauromorph footprints occur throughout the different sub-environments of the fluvial systems (Fig 13P). Similarly, Bernardi et al. [59] also suggested that the preferential habitats of archosauromorphs were the fluvial environments. Therefore, the Early Triassic archosauromorph radiation, increasing in diversity and final dominance [60, 63], may be linked to the widely extended and well-developed fluvial settings along Pangea after the end-Permian extinction event. On the instable Early Triassic ecosystems [3, 4], archosauromorph faunas were probably generalist [8, 11], taking advantage and occupying a widespread range of ecologic niches (see [3] for other groups). Key ecological innovations, such as those related to locomotion [49], permitted a rapid radiation and dominance of archosauromorphs, also hampering the radiation of other groups.

## Conclusions

The Triassic Pyrenean Basin is a key region of Western Tethys to understand the early Mesozoic non-marine evolution in terms of paleoenvironment and (ichno-) faunal diversity. The red-bed Buntsandstein deposits of the Catalan Pyrenees correspond to a fluvial setting evolving from high-energy braided systems to low-energy meandering and floodplain systems that infilled the depocenters generated during the Triassic rifting. In our case, the Buntsandstein facies are arranged as a fining-upwards sequence culminated by the marine transgression of the Muschelkalk facies. A relatively diverse fossil record is yielded in the red-beds, and tetrapod footprints are especially abundant. We identified an ichnoassemblage composed by *Prorotodactylus mesaxonichnus* isp. nov., cf. *Rotodactylus*, at least two different chirotheriid morphotypes, *Rhynchosauroides* cf. *schochardti* and two other *Rhynchosauroides* forms, an undetermined Morphotype A, and two types of swimming scratches corresponding to *Characichnos* isp. The potential trackmakers of *P*. *mesaxonichnus* isp. nov. are archosauriform euparkeriids, suggesting, together with the other ichnotaxa, an ichnofaunal homogeneity at least along Central Pangea. *Prorotodactylus* and *Rotodactylus* may characterize the late Early Triassic continental deposits. The trackmakers of all these ichnotaxa probably correspond to archosauromorphs, suggesting that this group became dominant in the instable terrestrial settings after the end-Permian mass extinction. Archosauromorphs may represent the main pull of vertebrate recovery that lead to the further ecosystem stabilization of the Middle Triassic, with a turnover to larger archosaurian faunas and the radiation of this lineage.

## Supporting information

S1 Text Sedimentology. Facies Description and Interpretation. Architectural Elements.
(DOCX)

S1 Table Track measurements of *Prorotodactylus mesaxonichnus* isp. nov. trackways. (DOCX)

S2 Table Trackway measurements of *Prorotodactylus mesaxonichnus* isp. nov. (DOCX)

S1 Fig Trackways of *Prorotodactylus mesaxonichnus* isp. nov. (A-C) Specimens from Erillcastell; dashed squares from A and C are correspond to manus-pes sets from Fig 7A and 7B, respectively. (D) Specimen from Buira.
(TIF)

S2 Fig Tracks of *Prorotodactylus mesaxonichnus* isp. nov. from Buira. (TIF)

S3 Fig Tracks of *Prorotodactylus mesaxonichnus* isp. nov. from Port del Cantó. Ichnites from sections IV (A) and VII (B).
(TIF)

S4 Fig Tracks of *Prorotodactylus mesaxonichnus* isp. nov. from Port del Cantó. (A) IPS-83740 from Argestues tracksite. (B-E) Isolated ichnites from Rubió tracksite. Note the relatively bad preservation of the ichnites, all in convex hyporelief, preserved in the bases of small meandering channels (facies *St*).
(TIF)

S5 Fig Tracks of chirotheriid morphotype indet. from Port del Cantó. (A) Manus-pes set (M-P). (B) Scratch-like track. (C) Partial track of IPS-83750.
(TIF)

S6 Fig Tracks of *Rhynchosauroides* morphotypes from Port del Cantó. (A-C) *Rhynchosauroides* cf. *schochardti*. (D) *Rhynchosauroides* isp. indet. 1. (E-G) *Rhynchosauroides* isp. indet.
(TIF)

S7 Fig Isolated and partially preserved tracks of the undetermined Morphotype A from Port del Cantó (section IV). (TIF)

## References

[1] Benton MJ, Tverdokhlebov VP, Surkov MV. Ecosystem remodelling among vertebrates at the Permian-Triassic boundary in Russia. Nature. 2004;432: 97–100. 10.1038/nature02950 15525988

[2] Benton MJ, Newell AJ. Impacts of global warming on Permo-Triassic terrestrial ecosystems. Gondwana Res. 2014;25: 1308–1337.

[3] Irmis RB, Whiteside JH. Delayed recovery of non-marine tetrapods after the end-Permian mass extinction tracks global carbon cycle. Proc R Soc B 2012;279: 1310–1318. 10.1098/rspb.2011.1895 22031757 PMC3282377

[4] Sahney S, Benton MJ. Recovery from the most profound mass extinction of all time. Proc R Soc B 2008;275: 759–765. 10.1098/rspb.2007.1370 18198148 PMC2596898

[5] Smith RMH, Botha-Brink J. Anatomy of a mass extinction: Sedimentological and taphonomic evidence for drought-induced die-offs at the Permo-Triassic boundary in the main Karoo Basin, South Africa. Palaeogeogr Palaeoclim Palaeoecol. 2014;396: 99–118.

[6] Bourquin S, Bercovici A, López-Gómez J, Diez JB, Broutin J, Ronchi A, Durand M, Arche A, Linol B, Amour F. The Permian-Triassic transition and the onset of Mesozoic sedimentation at the northwestern peri-Tethyan domain scale: Palaeogeographic maps and geodynamic implications. Palaeogeogr Palaeoclim Palaeoecol. 2011;299: 265–280.

[7] Borruel-Abadía V, López-Gómez J, De la Horra R, Galán-Abellán B, Barrenechea JF, Arche A, Ronchi A, Gretter N, Marzo M. Climate changes during the Early-Middle Triassic transition in the E. Iberian plate and their palaeogeographic significance in the western Tethys continental domain. Palaeogeogr Palaeoclim Palaeoecol 2015;440: 671–689.

[8] Mujal E, Gretter N, Ronchi A, López-Gómez J, Falconnet J, Diez JB, et al Constraining the Permian/Triassic transition in continental environments: Stratigraphic and paleontological record from the Catalan Pyrenees (NE Iberian Peninsula). Palaeogeogr Palaeoclim Palaeoecol. 2016;445: 18–37.

[9] Klein H, Voigt S, Hminna A, Saber H, Schneider JW, Hmich D. Early Triassic Archosaur-Dominated Footprint Assemblage from the Argana Basin (Western High Atlas, Morocco). Ichnos. 2010;17(3): 215–227.

[10] Tourani A, Benaouiss N, Gand G, Bourquin S, Jalil N-E, Broutin J, et al Evidence of an Early Triassic age (Olenekian) in Argana Basin (High Atlas, Morocco) based on new chirotherioid traces. C R Palevol. 2010;9: 201–208.

[11] Petti FM, Bernardi M, Kustatscher E, Renesto S, Avanzini M. Diversity of continental tetrapods and plants in the Triassic of the Southern Alps: Ichnological, paleozoological and paleobotanical evidence. N M Mus Nat Hist Sci Bull. 2013;61: 458–484.

[12] Krainer K, Lucas SG, Ronchi A. Tetrapod footprints from the Alpine Buntsandstein (Lower Triassic) of the Drau Range (Eastern Alps, Austria). Jahrb Geol B-A. 2012;152(1–4): 205–212.

[13] Ptaszyński T. Lower Triassic vertebrate footprints from Wióry, Holy Cross Mountains, Poland. Acta Palaeontol Pol. 2000;45(2): 151–194.

[14] Niedźwiedzki G, Ptaszyński P. Large Chirotheriidae tracks in the Early Triassic of Wióry, Holy Cross Mountains, Poland. Acta Geol Pol. 2007;57(3): 325–342.

[15] Brusatte SL, Niedźwiedzki G, Butler RJ. Footprints pull origin and diversification of dinosaur stem lineage deep into Early Triassic. Proc R Soc B. 2011;278: 1107–1113. 10.1098/rspb.2010.1746 20926435 PMC3049033

[16] Klein H, Niedźwiedzki G. Revision of the Lower Triassic tetrapod ichnofauna from Wióry, Holy Cross Mountains, Poland. N M Mus Nat Hist Sci Bull. 2012;59: 1–62.

[17] Fichter J, Kunz R. “Dinosauromorph” tracks from the Middle Buntsandstein (Early Triassic: Olenekian) of Wolfhagen, northern Hesse, Germany. Comunicações Geológicas. 2013;100(1): 81–88.

[18] Fichter J, Kunz R. Eine Tetrapoden-Fährtenvergesellschaftung im Mittleren Buntsandstein (frühe Trias: Olenekium) von Wolfhagen, Nordhessen. Z Dt Ges Geowiss. 2015;166(3): 253–273.

[19] Niedźwiedzki G, Brusatte SL, Butler RJ. *Prorotodactylus* and *Rotodactylys* tracks: an ichnological record of dinosauromorphs from the Early-Middle Triassic of Poland In: Nesbit SJ, Desojo JB, Irmis RB, editors. Anatomy, Phylogeny and Palaeobiology of Early Archosaurs and their Kin. Geological Society Special Publications 379, London; 2013 pp. 319–351.

[20] Klein H, Lucas SG. Review of the tetrapod ichnofauna of the Moenkopi formation/group (Early-Middle Triassic) of the American Southwest. N M Mus Nat Hist Sci Bull. 2010;50: 1–167.

[21] Lovelace DM, Lovelace SD. Paleoenvironments and paleoecology of a Lower Triassic invertebrate and vertebrate ichnoassemblage from the Red Peak Formation (Chugwater Group), Central Wyoming. Palaios. 2012;27: 636–657.

[22] Thomson TJ, Droser ML. Swimming reptiles make their mark in the Early Triassic: Delayed ecologic recovery increased the preservation potential of vertebrate swim tracks. Geology. 2015;43(3): 215–218.

[23] Mey PHW, Nagtegaal PJC, Roberti KJ, Hartevelt JJA. Lithostratigraphic subdivision of post-Variscan deposits in the South-Central Pyrenees, Spain. Leidse Geol Meded. 1968;41: 153–220.

[24] Nagtegaal PJC. Sedimentology, paleoclimatology, and diagenesis of post-Hercynian continental deposits in the south-central Pyrenees, Spain. Leidse Geol Meded. 1969;42: 143–238.

[25] Séguret M. Etude tectonique des nappes et séries décollés de la partie centrale du versant sud des Pyrenées. Caractère synsédimentaire, rôle de la compression et de la gravité. PhD thesis, Université de Montpellier, Publ. USTELA. Série Géol Struct. 1972;2: 1–210.

[26] Zwart HJ. The Geology of Central Pyrenees. Leidse Geol Meded. 1979;50: 1–74.

[27] Speksnijder A. Anatomy of a strike-slip fault controlled sedimentary basin, Permian of the southern Pyrenees, Spain. Sediment Geol. 1985;44: 179–223.

[28] Saura E. Anàlisi estructural de la zona de les Nogueres Pirineus Centrals. Ph.D Thesis, Universitat Autònoma de Barcelona, 2004. http://www.tdx.cat/handle/10803/3438

[29] Saura E, Teixell A. Inversion of small basins: effects on structural variations at the leading edge of the Axial Zone antiformal stack (Southern Pyrenees, Spain). J Struct Geol. 2006;28: 1909–1920.

[30] Gretter N, Ronchi A, López-Gómez J, Arche A, De la Horra R, Barrenechea JF, Lago M. The Late Palaeozoic-Early Mesozoic from the Catalan Pyrenees (Spain): 60 Myr of environmental evolution in the frame of the western peri-Tethyan palaeogeography. Earth-Sci Rev. 2015;150: 679–708.

[31] Fortuny J, Bolet A, Sellés AG, Cartanyà J, Galobart À. New insights on the Permian and Triassic vertebrates from the Iberian Peninsula with emphasis on the Pyrenean and Catalonian basins. J Iber Geol. 2011;37(1): 65–86.

[32] Torsvik TH, Cocks LRM. Gondwana from top to base in space and time. Gondwana Res. 2013;24: 999–1030.

[33] Miall AD. Architectural-element analysis: a new method of facies analysis applied to fluvial deposits. Earth-Sci Rev. 1985;22: 261–308.

[34] Miall AD. Alluvial deposits In: Walker RG, James NP, editors. Facies Models. Geological Association of Canada Publications, Ontario; 1992 pp. 119–142.

[35] Haubold H. Die Tetrapodenfährten des Buntsandsteins in der Deutschen Demokratischen Republik und in Westdeutschland und ihre Äquivalente in der gesamten Trias. Paläontologische Abhandlungen, Abteilung A Paläozoologie. 1971: 395–548.

[36] Haubold H. Ichnia Amphibiorum et Reptiliorum fossilium. Encyclopedia of Paleoherpetology 18. Gustav Fischer Verlag, Stuttgart and Portland-USA; 1971.

[37] Leonardi G. Glossary and manual of tetrapod footprint palaeoichnology. Departamento Nacional de Produção Mineral, Brasilia; 1987.

[38] Mallison H, Wings O. Photogrammetry in paleontology—A practical guide. Journal of Paleontological Techniques. 2014;12: 1–31.

[39] Mujal E, Fortuny J, Oms O, Bolet A, Galobart À, Anadón P. Palaeoenvironmental reconstruction and early Permian ichnoassemblage from the NE Iberian Peninsula (Pyrenean Basin). Geol Mag. 2016;153(4): 578–600.

[40] Calvet F, Solé de Porta N, Salvany JM. Cronoestratigrafía (Palinología) del Triásico sudpirenaico y del Pirineo Vasco-Cantábrico. Acta Geol Hisp. 1993;28: 33–48.

[41] Demathieu G. Une ichnofaune du Trias Moyen du Bassin de Lodève (Hérault, France). Annales de Paléontologie. 1984;70(4): 247–273.

[42] Carrano MT, Wilson JA. Taxon distributions and the tetrapod track record. Paleobiology. 2001;27(3): 564–582.

[43] Nesbitt SJ. The early evolution of archosaurs: relationships and the origin of major clades. Bull Am Mus Nat Hist. 2011;352; 1–292.

[44] Ezcurra MD. The phylogenetic relationships of basal archosauromorphs, with an emphasis on the systematics of proterosuchian archosauriforms. PeerJ. 2016;4: e1778 10.7717/peerj.1778 27162705 PMC4860341

[45] Langer MC, Nesbitt SJ, Bittencourt JS, Irmis RB. Non-dinosaurian Dinosauromorpha In: Nesbit SJ, Desojo JB, Irmis RB, editors. Anatomy, Phylogeny and Palaeobiology of Early Archosaurs and their Kin. Geological Society Special Publications 379, London; 2013 pp. 157–186.

[46] Padian K. The problem of dinosaur origins: integrating three approaches to the rise of Dinosauria. Earth Environ Sci Trans R Soc Edinb. 2013;103: 1–20.

[47] Ewer RF. The anatomy of the thecodont reptile *Euparkeria capensis* Broom. Phil Trans R Soc Lond B. 1965;248: 379–435.

[48] Sookias RB, Butler RJ. Euparkeriidae In: Nesbit SJ, Desojo JB, Irmis RB, editors. Anatomy, Phylogeny and Palaeobiology of Early Archosaurs and their Kin. Geological Society Special Publications 379, London; 2013 pp. 35–48.

[49] Sookias RB. The relationships of the Euparkeriidae and the rise of Archosauria. R Soc open sci. 2016;3: 150674 10.1098/rsos.150674 27069658 PMC4821269

[50] Peabody FE. Reptile and amphibian trackways from the Moenkopi Formation of Arizona and Utah. University of California Publications, Bulletin of the Department of Geological Sciences. 1948;27: 295–468.

[51] Gand G, Demathieu G, Montenat C. Les traces de pas d'amphibiens, de dinosaures et autres reptiles du Mésozoïque français: Inventaire et interprétations. Palaeovertebrata 2007;1–4: 1–149.

[52] Klein H, Lucas SG. Tetrapod footprints—their use in biostratigraphy and biochronology of the Triassic. Geol Soc Spec Publ. 2010;334: 419–446.

[53] Klein H, Niedźwiedzki G, Voigt S, Lagnaoui A, Hminna A, Saber H, et al The tetrapod ichnogenus Protochirotherium Fichter and Kunz 2004, a characteristic Early Triassic morphotype of Central Pangea. Ichnos. 2013;20(1): 24–30.

[54] Haubold H, Klein H. Chirotherien und Grallatoriden aus der Unteren bis Oberen Trias Mitteleuropas und die Entstehung der Dinosauria. Hallesches Jahrb Geowiss. 2002;24: 1–22.

[55] Avanzini M, Bernardi M, Nicosia U. The Permo-Triassic tetrapod faunal diversity in the Italian Southern Alps In: Dar IA, editor. Earth and Environmental Sciences. InTech; 2011 pp. 591–608.

[56] Whyte MA, Romano M. A dinosaur ichnocoenosis from the Middle Jurassic of Yorkshire, UK. Ichnos. 2001;8: 223–234.

[57] Klein H, Haubold H. Archosaur footprints—Potential for biochronology of Triassic continental sequences. N M Mus Nat Hist Sci Bull. 2007;641: 120–130.

[58] Ezcurra MD, Scheyer TM, Butler RJ. The Origin and Early Evolution of Sauria: Reassessing the Permian Saurian Fossil Record and the Timing of the Crocodile-Lizard Divergence. PLoS ONE. 2014;9(2): e89165 10.1371/journal.pone.0089165 24586565 PMC3937355

[59] Bernardi M, Klein H, Petti FM, Ezcurra MD. The Origin and Early Radiation of Archosauriforms: Integrating the Skeletal and Footprint Record. PLoS ONE. 2015;10(6): e0128449 10.1371/journal.pone.0128449 26083612 PMC4471049

[60] Pinheiro FL, França MAG, Lacerda MB, Butler RJ, Schultz CL. An exceptional skull from South America and the origins of the archosauriform radiation. Sci Rep 2016;6: 22817 10.1038/srep22817 26965521 PMC4786805

[61] Haubold H. Tracks of the Dinosauromorpha from the Early Triassic In: Bachmann GH, Lerche I, editors. Triassic: Zentralblatt für Geologie und Paläontologie, part 1, 1998 (7–8); 1999 pp. 783–795.

[62] Ezcurra MD, Butler RJ. Taxonomy of the proterosuchid arhosauriforms (Diapsida: Archoauromorpha) from the earliest Triassic of South Africa, and implications for the early archosauriform radiation. Palaeontology. 2015;58(1): 141–170.

[63] Sookias RB, Butler RJ, Benson RBJ. Rise of dinosaurs reveals major body-size transitions are driven by passive processes of trait evolution. Proc R Soc B. 2012;279: 2180–2187. 10.1098/rspb.2011.2441 22298850 PMC3321709

