# Supplementary material for: An archosauromorph dominated ichnoassemblage in fluvial settings from the late Early Triassic of the Catalan Pyrenees (NE Iberian Peninsula)
Source: PLoS One. 2017 Apr 19;12(4):e0174693. doi: 10.1371/journal.pone.0174693 (PMC5396874; doi:10.1371/journal.pone.0174693)
Supplement: S1 Text — Facies Description and Interpretation. Architectural Elements. (DOCX) [file pone.0174693.s001.docx]

**An Archosauromorph Dominated Ichnoassemblage in Fluvial Settings from the late Early Triassic of the Catalan Pyrenees (NE Iberian Peninsula)**

Eudald Mujal*, Josep Fortuny, Arnau Bolet, Oriol Oms, José Ángel López

*Corresponding author: Departament de Geologia, Universitat Autònoma de Barcelona, E-08193 Bellaterra, Spain; e-mail: eudald.mujal@gmail.com

**S1 Text. Sedimentology.**

**Facies Description and Interpretation**

*Facies Gh:* They are composed of clast-supported conglomerates, with subordinate matrix-supported levels, generally massive, with crude stratification. The matrix is composed of coarse to very coarse sandstone. Bedding is up to 50 cm thick and is often arranged as fining-upwards sequences and present erosive bases with flow structures such as flute casts. They are mainly located in the conglomerate unit (associated with facies *Gmpt*, *St* and *Sp*) and occasionally in the shale and sandstones unit (associated with *St*). These facies are interpreted as basal lag deposit or the frontal part of a channel bar.

*Facies Gmpt:* They are composed of clast- to matrix-supported conglomerates, with crude planar stratification, planar cross stratification, or trough cross stratification, and levels with imbricated pebbles. Sets thickness range from 50 to 90 cm, and cosets are up to 2 m. Matrix is composed of coarse to very coarse sandstone. These facies occasionally grade upwards to facies *St* and *Sp*. *Gmpt* is interpreted as longitudinal bars from high-flow braided streams, as minor channel fills, or as lag deposits when associated with sandstone facies.

Both facies *Gh* and *Gmpt* are oligomictic in composition (reworked dyke quartz mostly rounded to sub-rounded) with occasional black and greenish lydite and slate fragments. Clasts average size is of 10 cm (with pebbles up to 15 cm) in some beds, and of 2-4 cm (with pebbles up to 6-7 cm) in others. These facies are mostly the result of high-flow braided fluvial systems, and occasionally correspond to minor lag deposits of meandering fluvial systems.

*Facies St:* They are composed of fine- to medium-grained sandstones (rarely coarse to very coarse), with trough cross stratification. Beds present lenticular geometry, but in some cases are tabular. They build up fining-upwards sequences ranging from 10 to 90 cm thick (commonly of 20­­-30 cm), and often grade to facies *Sp*. Cosets are up to 1.5 m thick. Sets are bounded by facies *Fsc* or partially eroded by other *St* and *Sp* sets, the latter corresponding to low- to medium-angle dipping surfaces (reactivation surfaces). Soft (mudstone) pebbles are present in some levels. In some cases clasts as those from *Gmpt* are found as lag deposits. The facies *St* are interpreted as subaqueous dunes associated to channel fills, minor bars and sand flats of braided systems (conglomerate unit), and as point bars of meandering fluvial systems (shale and sandstones unit).

*Facies Sp:* They are composed of fine- to medium-grained sandstones (rarely coarse to very coarse), with planar cross stratification. Sets resemble those of facies *St*, but generally display a tabular geometry with subordinated lenticular bodies. In the shale and sandstones Unit often grades to facies *Sr*, *Sh* and *Fsc* (fining upwards sequences). The facies *Sp* are interpreted as linguoid bars and sand flats of braided systems (conglomerate unit) and transverse bars of meandering systems (shale and sandstones unit).

*Facies Sr:* They are composed of fine- to very fine-grained sandstones with abundant climbing ripples, and also flow and wave ripples. Beds are of 10 to 30 cm thick, in sets of tabular and channel geometry. Cosets are up to 1.5 m thick, and are commonly grading from *Sp* to *Sl* and *Fsc* (fining upwards sequences), and also present interbedded *Fl*. The upper part of some bodies are burrowed. The facies *Sr* are interpreted as the result of low regime flows, corresponding to the upper part of the scroll bar of meandering systems.

*Facies Sh:* They are composed of fine- to very fine-grained sandstones with parallel laminations. Bodies are of tabular geometry and grouped in sets up to 20 cm thick. Greenish reduction mottles are common. The facies *Sh* are interpreted as planar beds from upper flow regime.

*Facies Sl:* They are composed of fine- to very fine-grained sandstones with climbing and flow ripples. Sets (up to 30 cm thick) and cosets (up to 1 m thick) are bounded by low- to medium-angle inclined planes (reactivation surfaces, similar to those of *St* and *Sp*, but finer grained). These facies are often associated with facies *Fl*, forming both fining and coarsening upwards sequences. Isolated lenticular beds with erosive bases also occur. The facies *Fl* are interpreted as scour fill deposits or washed-out dunes.

*Facies Se:* They are composed of fine- to coarse-grained sandstones with crude trough cross stratification and soft pebbles. Bodies present strong erosive bases and are laterally discontinuous. The thickness varies from 15 to 35 cm. Mud-cracked and bioturbated surfaces occur, denoting occasional energetic episodes with long subaerial exposure. The facies *Se* are interpreted as scour fill deposits.

*Facies Fl:* They are composed of very fine-grained (occasionally fine-grained) sandstones and siltstones with fine lamination and flow ripples. They are found as deposits of 6-8 m thick, interbedded with facies *Sl* and *Fsc*. These facies often grade from facies *Sp* and *Sr*. The facies *Fl* are interpreted as overbank deposits associated to meandering channels.

*Facies Fsc:* They are composed of siltstones and claystones, and very fine-grained sandstones. They are massive or fine laminated. Thickness is up to 7 m. The facies *Fsc* are the most abundant in the shale unit, and are always present in transition to the Muschelkalk facies. The facies *Fsc* are interpreted as floodplain deposits.

*Facies Fm:* They are composed of siltstones and claystones with mud-cracked surfaces. Bodies are interbedded with facies *Fsc* and *Fl*, and are often eroded by deposits of facies *Sp* and *Sr*. Set thickness is about 0.5 m, but sequences up to 4 m also exist. Some levels present edaphic carbonate nodules (transition to facies *P*) and reduction mottles. The facies *Fm* are interpreted as overbank or drape deposits that underwent subaerial exposure.

*Facies Fr:* They are composed of massive siltstones and claystones with root traces, bioturbations and rain drops. Small greenish reduction mottles as those of facies *Sh* are common. The facies *Fr* are interpreted as muddy floodplain deposits.

*Facies P:* They are composed of massive siltstones and claystones. Two main types of intervals are distinguished: (1) levels with carbonate nodules, where nodules are generally small (0.5-1 cm of diameter), but occasionally (i.e., in the upper part of the Buntsandstein succession from Erillcastell locality) they are larger, reaching 4-5 cm of diameter, purple-yellowish colored and with little mudstone matrix; (2) hardened siltstone and claystone intervals (associated to facies *Fsc*) displaying large green reduction mottles that build up continuous levels parallel to stratification, these levels may preserve slickensides and root traces in the uppermost part. The facies *P* are interpreted as paleosols, resulting from pedogenic processes, developed in overbank deposits from floodplain systems.

**Architectural Elements**

*Element CH1:* It is composed of conglomerates and coarse-grained sandstone bodies, constituted by facies *Gh*, *Gmpt*, *St* and *Sp*. It is located at the basal conglomerate unit and interpreted as channels and longitudinal bars of braided river systems.

*Element CH2:* It is composed of medium-fine grained sandstone bodies, constituted by facies *St*, *Sp*, *Sr*, and occasionally *Gh*. It is located at the shale and sandstones unit and interpreted as channels of small river streams associated to occasional events.

*Element GB:* It is composed of conglomerate and very coarse deposits, constituted by facies *Gh* and *Gmpt*. It is located at the basal conglomerate unit and interpreted as gravel bars and bedforms, usually of tabular geometry.

*Element SB:* It is composed of coarse to very fine sandstones, constituted by facies *St*, *Sp*, *Sr*, *Sh*, *Sl* and *Se*, and sometimes grading to facies *Fsc*. It is located at the upper part of the conglomerate unit, where corresponds to channel fills or minor bars of braided systems. It is also located at the shale and sandstones unit as sandy bedforms associated to lateral accretions (facies association *St*-*Sp*), thus corresponding to upper part of the point bars from meandering systems, but also as crevasse splay, minor bars and channel fill deposits.

*Element LA:* It is composed of medium to fine-very fine sandstones, constituted by facies *St* and *Sp*. It is located at the shale and sandstones unit, corresponding to lateral accretions of meandering point bars.

*Element LS:* It is mostly composed of fine sandstones, constituted by facies *Sh* and *Sl*, and minor facies *Sp* and *Sr*. It is located at the shale and sandstones unit and the shale unit, corresponding to laminated sand sheet deposits.

*Element OF:* It is composed of claystones and siltstones and subordinated very fine-to fine-grained sandstones, constituted by facies *Sl*, *Fr*, *Fl*, *Fm* and *Fsc*. It is located at the shale and sandstones unit and the shale unit, corresponding to overbank fines deposits that may fill abandoned channels.
