# Supplementary material for: An archosauromorph dominated ichnoassemblage in fluvial settings from the late Early Triassic of the Catalan Pyrenees (NE Iberian Peninsula)
Source: PLoS One. 2017 Apr 19;12(4):e0174693. doi: 10.1371/journal.pone.0174693 (PMC5396874; doi:10.1371/journal.pone.0174693)
Supplement: S1 Table — (DOCX) [file pone.0174693.s002.docx]

**An Archosauromorph Dominated Ichnoassemblage in Fluvial Settings from the late Early Triassic of the Catalan Pyrenees (NE Iberian Peninsula)** Eudald Mujal*, Josep Fortuny, Arnau Bolet, Oriol Oms, José Ángel López *Corresponding author: Departament de Geologia, Universitat Autònoma de Barcelona, E-08193 Bellaterra, Spain; e-mail: eudald.mujal@gmail.com

**S1 Table. Track measurements of the *Prorotodactylus mesaxonichnus* isp. nov. trackways.** Values resumed in Table 1.

| **Trackway holotype**  **(IPS-93870)** | **Length** | **Width** | **Digit I** | **Digit II** | **Digit III** | **Digit IV** | **Digit V** | **Length I-IV** | **Width I-IV** | **Div. I-II** | **Div. II-III** | **Div. III-IV** | **Div. IV-V** | **Div. II-IV** | **Div. I-IV** | **Div. I-V** |
| --- | --- | --- | --- | --- | --- | --- | --- | --- | --- | --- | --- | --- | --- | --- | --- | --- |
| **Manus #1** | **50.288** | 30.503 | **12.973** | 23.128 | 33.219 | 27.521 | **12.666** | 41.791 | 24.658 | 21.132 | 14.763 | 10.840 | 41.393 | 25.603 | 46.735 | 88.128 |
| **Pes #1** | - | - | - | - | **42.07** | - | - | - | - | - | - | - | - | - | - | - |
| **Manus #2** | 34.819 | 32.712 | 11.011 | 18.575 | 22.342 | 19.788 | **14.249** | 30.982 | 26.369 | 14.838 | 14.562 | 21.858 | 19.705 | 36.42 | 51.258 | 70.963 |
| **Pes #2** | **50.125** | - | 21.144 | - | 30.598 | 26.667 | - | 41.778 | 44.263 | - | - | 15.068 | - | - | 46.861 | - |
| **Manus #3** | 40.672 | 28.741 | **12.233** | 18.490 | 25.646 | 19.947 | 14.792 | 38.059 | 22.547 | 32.835 | 16.999 | 8.479 | 33.371 | 25.478 | 58.313 | 91.684 |
| **Pes #3** | **62.313** | - | 23.037 | 30.503 | 38.499 | 36.149 | - | 53.121 | 44.388 | 18.564 | 18.968 | 9.841 | - | 28.809 | 47.373 | - |
| **Manus #4** | 43.782 | 31.483 | 13.539 | 18.49 | 23.259 | 21.908 | 13.074 | 40.301 | 22.848 | 25.241 | 17.236 | 5.584 | 30.629 | 22.82 | 48.061 | 78.69 |
| **Pes #4** | 61.464 | - | 25.461 | 32.144 | 40.21 | 35.533 | - | 50.873 | 53.513 | 18.865 | 16.682 | 16.977 | - | 33.659 | 52.524 | - |
| **Manus #5** | **26.672** | - | 10.384 | 14.286 | 16.954 | 10.781 | **-** | 24.124 | 18.981 | 11.138 | 12.703 | 17.489 | - | 30.192 | 41.33 | - |
| **Pes #5** | **60.438** | - | 27.269 | 36.850 | 39.889 | 37.657 | - | 53.923 | 48.919 | 17.617 | 19.492 | 16.407 | - | 35.899 | 53.516 | - |
| **Manus #6** | 42.285 | 31.815 | 12.580 | 16.867 | 24.391 | 23.338 | 14.375 | 38.135 | 22.262 | 21.625 | 14.682 | 9.038 | 44.892 | 23.72 | 45.345 | 90.237 |
| **Pes #6** | **54.277** | - | 23.612 | 32.175 | 36.415 | 30.583 | - | 43.745 | 48.529 | 12.947 | 17.326 | 15.057 | - | 32.383 | 45.33 | - |
| **Manus #7** | 48.226 | 35.813 | 14.831 | 20.404 | 27.802 | 25.904 | 18.878 | 40.111 | 26.957 | 25.453 | 21.678 | 9.269 | 39.389 | 30.947 | 56.4 | 95.789 |
| **Pes #7** | **67.09** | - | 29.98 | 36.774 | 43.949 | 42.295 | - | 55.961 | 44.776 | 14.509 | 18.151 | 18.485 | - | 36.636 | 51.145 | - |
| **Manus #8** | 45.491 | - | 11.131 | 19.246 | 27.726 | 25.85 | - | 36.929 | 26.36 | 27.441 | 16.674 | 16.704 | - | 33.378 | 60.819 | - |
| **Pes #8** | **64.948** | - | 28.068 | 37.095 | 42.096 | 38.564 | - | 57.781 | 39.142 | 9.297 | 10.03 | 11.04 | - | 21.07 | 30.367 | - |
| **Mean Manus tracks** | 41.529 | 31.845 | 12.335 | 18.686 | 25.167 | 21.880 | 14.672 | 36.304 | 23.873 | 22.463 | 16.162 | 12.408 | 34.897 | 28.570 | 51.033 | 85.915 |
| **Mean Pes tracks** | 60.094 | - | 25.510 | 34.257 | 39.216 | 35.350 | - | 51.026 | 46.219 | 15.300 | 16.775 | 14.696 | - | 31.409 | 46.731 | - |

Units in mm and degrees. Highlighted values are estimated.

**S1 Table.** *(continued)*

| **Trackway**  **Paratype**  **(IPS-93867)** | **Length** | **Width** | **Digit I** | **Digit II** | **Digit III** | **Digit IV** | **Digit V** | **Length I-IV** | **Width I-IV** | **Div. I-II** | **Div. II-III** | **Div. III-IV** | **Div. IV-V** | **Div. II-IV** | **Div. I-IV** | **Div. I-V** |
| --- | --- | --- | --- | --- | --- | --- | --- | --- | --- | --- | --- | --- | --- | --- | --- | --- |
| **Manus #1** | 27.632 | 19.582 | 8.480 | 10.573 | 16.179 | 15.587 | 10.506 | 24.224 | 14.629 | 36.870 | 23.420 | 8.228 | 7.585 | 31.648 | 68.518 | 76.103 |
| **Pes #1** | 47.282 | 31.481 | 22.052 | 24.206 | 33.511 | 29.499 | **13.609** | 42.541 | 22.627 | 15.018 | 9.421 | 6.919 | 25.488 | 16.340 | 31.358 | 56.846 |
| **Manus #2** | 28.860 | **18.449** | - | 12.032 | 14.295 | 13.559 | 11.806 | - | **13.298** | - | 13.626 | 14.826 | 37.679 |  | - | - |
| **Pes #2** | - | - | 19.237 | 22.052 | 24.641 | - | - | - | - | 14.364 | 12.274 | - | - | - | - | - |
| **Manus #3** | - | - | - | - | **15.360** | 15.799 | 13.052 | - | - | - | - | 12.130 | 15.489 | - | - | - |
| **Pes #3** | 42.394 | 24.367 | 19.593 | 25.327 | 29.032 | 28.284 | 16.731 | 38.861 | 17.200 | 11.591 | 9.783 | 8.151 | 15.905 | 17.934 | 29.525 | 45.430 |
| **Manus #4** | 21.368 | 19.58 | 7.639 | 10.483 | 13.167 | 12.739 | 8.336 | 16.101 | 13.782 | 21.498 | 23.523 | 11.206 | 45.772 | 34.729 | 56.227 | 101.999 |
| **Manus #5** | 23.165 | 18.325 | 7.498 | 8.895 | 12.507 | 12.794 | 8.958 | 18.24 | 13.746 | 21.267 | 7.243 | 11.958 | 31.75 | 19.201 | 40.468 | 72.218 |
| **Pes #5** | 36.542 | - | 14.265 | 15.540 | 18.977 | 18.104 | - | 31.515 | 17.706 | 15.299 | 14.774 | 10.78 | - | 25.554 | 40.853 | - |
| **Manus #6** | 23.439 | 15.649 | 6.041 | 8.889 | 15.289 | 11.635 | 6.942 | 20.122 | 12.739 | 40.409 | 15.377 | 10.107 | 47.491 | 25.484 | 65.893 | 113.384 |
| **Pes #6** | **32.507** | 26.111 | 12.087 | 14.327 | 20.008 | 18.325 | - | 25.649 | 20.576 | 14.087 | 13.666 | 15.748 | **44.105** | 29.414 | 43.501 | 87.606 |
| **Manus #7** | 24.425 | 17.349 | **6.704** | 8.891 | 15.063 | **14.489** | 8.143 | 22.602 | 13.378 | 16.905 | 11.937 | 7.655 | 20.716 | 19.592 | 36.497 | 57.213 |
| **Pes #7** | **46.521** | 29.446 | 12.775 | 18.663 | 24.942 | 24.01 | - | 33.772 | 23.692 | 11.868 | 13.302 | 10.789 | **38.006** | 24.091 | 35.959 | 73.965 |
| **Manus #9** | 23.168 | **23.105** | 10.275 | 10.026 | 12.348 | 9.300 | **8.480** | 16.167 | 19.582 | 15.183 | 9.802 | 11.763 | 19.896 | 21.565 | 36.748 | 56.644 |
| **Pes #9** | 40.281 | - | 19.201 | 20.243 | 23.487 | 21.526 | - | 32.824 | 31.803 | 9.868 | 10.564 | 10.099 | - | 20.663 | 30.531 | - |
| **Mean Manus tracks** | 24.580 | 18.863 | 7.773 | 9.970 | 14.276 | 13.238 | 9.528 | 19.576 | 14.451 | 25.355 | 14.990 | 10.984 | 28.297 | 25.370 | 50.725 | 79.594 |
| **Mean Pes tracks** | 40.921 | 27.851 | 17.030 | 20.051 | 24.943 | 23.291 | 15.170 | 34.194 | 22.267 | 13.156 | 11.969 | 10.414 | 30.876 | 22.333 | 35.288 | 65.962 |

**S1 Table.** *(continued)*

| **Trackway**  **S1A Fig** | **Length** | **Width** | **Digit I** | **Digit II** | **Digit III** | **Digit IV** | **Digit V** | **Length I-IV** | **Width I-IV** | **Div. I-II** | **Div. II-III** | **Div. III-IV** | **Div. IV-V** | **Div. II-IV** | **Div. I-IV** | **Div. I-V** |
| --- | --- | --- | --- | --- | --- | --- | --- | --- | --- | --- | --- | --- | --- | --- | --- | --- |
| **Manus #1** | **25.284** | - | **9.112** | 15.010 | 17.786 | 15.617 | - | 22.502 | 16.398 | 36.777 | 5.603 | 6.341 | - | 11.944 | 48.721 | - |
| **Pes #1** | **54.793** | - | 20.952 | 25.119 | 30.562 | 29.284 | - | 46.212 | 32.872 | 9.271 | 12.218 | 12.319 | - | 24.537 | 33.808 | - |
| **Manus #2** | **40.339** | 27.977 | 12.485 | 19.157 | 22.099 | 19.769 | **14.487** | 28.922 | 21.454 | 16.274 | 14.481 | 14.717 | 38.246 | 29.198 | 45.472 | 83.718 |
| **Pes #2** | - | - | - | - | - | - | - | - | - | 17.745 | - | - | - | - | - | - |
| **Manus #3** | **32.473** | 24.321 | 9.672 | 17.090 | 21.259 | 19.504 | **13.437** | 32.802 | 20.048 | 26.630 | 12.757 | 12.010 | 29.281 | 24.767 | 51.397 | 80.678 |
| **Pes #3** | - | - | - | - | - | - | - | - | - | - | - | - | - | - | - | - |
| **Manus #4** | **29.827** | - | 11.801 | 15.300 | 19.295 | 15.918 | - | 26.565 | 19.054 | 17.494 | 14.273 | 15.555 | - | 29.828 | 47.322 | - |
| **Pes #4** | **45.138** | - | 21.973 | 26.750 | 31.353 | 27.126 | - | 39.527 | 38.254 | 10.876 | 12.370 | 20.045 | - | 32.415 | 43.291 | - |
| **Manus #5** | 34.052 | 24.752 | 9.044 | 13.620 | 20.983 | 20.077 | 11.338 | 28.167 | 18.940 | 23.867 | 15.129 | 11.443 | 33.331 | 26.572 | 50.439 | 83.77 |
| **Pes #5** | - | - | 18.663 | - | - | - | - | - | - | - | - | - | - | - | - | - |
| **Manus #6** | 36.693 | 29.389 | 10.787 | 16.257 | 20.563 | 18.932 | 14.34 | 27.55 | 22.543 | 18.915 | 16.198 | 11.997 | 41.389 | 28.195 | 47.11 | 88.499 |
| **Pes #6** | **56.013** | - | 21.128 | 33.566 | 35.591 | 35.042 | - | 47.553 | 40.293 | 17.309 | 19.888 | 20.917 | - | 40.805 | 58.114 | - |
| **Mean Manus tracks** | 33.111 | 26.610 | 10.484 | 16.072 | 20.331 | 18.303 | 13.401 | 27.751 | 19.740 | 23.326 | 13.074 | 12.011 | 35.562 | 25.084 | 48.410 | 84.166 |
| **Mean Pes tracks** | 51.981 | - | 20.679 | 28.478 | 32.502 | 30.484 | - | 44.431 | 37.140 | 13.800 | 14.825 | 17.760 | - | 32.586 | 45.071 | - |

**S1 Table.** *(continued)*

| **Trackway**  **S1B Fig** | **Length** | **Width** | **Digit I** | **Digit II** | **Digit III** | **Digit IV** | **Digit V** | **Length I-IV** | **Width I-IV** | **Div. I-II** | **Div. II-III** | **Div. III-IV** | **Div. IV-V** | **Div. II-IV** | **Div. I-IV** | **Div. I-V** |
| --- | --- | --- | --- | --- | --- | --- | --- | --- | --- | --- | --- | --- | --- | --- | --- | --- |
| **Manus #1** | **43.338** | - | - | - | - | 24.479 | 17.453 | - | - | - | - | - | 25.816 | - | - | - |
| **Pes #1** | 55.418 | - | 26.530 | 34.682 | 39.937 | 36.532 | - | 56.702 | 32.970 | 12.698 | 10.445 | 13.444 | - | 23.889 | 36.587 | - |
| **Manus #2** | 35.368 | **25.269** | 12.434 | 13.766 | 18.246 | 16.956 | - | 24.997 | 21.099 | 12.760 | 10.764 | 11.835 | - | 22.599 | 35.359 | - |
| **Pes #2** | - | - | - | - | - | - | - | - | - | **11.635** | - | - | - | - | - | - |
| **Manus #3** | 32.015 | 23.835 | 11.748 | 15.071 | 20.368 | 18.103 | 9.975 | 22.901 | 19.168 | 13.370 | 21.842 | 12.273 | 24.332 | 34.115 | 47.485 | 71.817 |
| **Pes #3** | 51.493 | 46.895 | 26.014 | 28.295 | 34.344 | 31.890 | - | 48.412 | 33.495 | 13.076 | 9.165 | 7.988 | **57.596** | 17.153 | 30.229 | 87.825 |
| **Manus #4** | 32.235 | 24.214 | 11.376 | 18.538 | 23.673 | 21.496 | **12.163** | 25.754 | 23.086 | 20.783 | 16.406 | 14.799 | **15.723** | 31.205 | 51.988 | 67.711 |
| **Pes #4** | 42.428 | - | 21.805 | 26.647 | 30.858 | **30.505** | - | 40.738 | 39.075 | 18.119 | 21.135 | 16.878 | - | 38.013 | 56.132 | - |
| **Manus #5** | **30.502** | 22.360 | **9.111** | 14.123 | 20.970 | 19.520 | **11.579** | 27.550 | 16.507 | 25.275 | 15.571 | 10.622 | 35.530 | 26.193 | 51.468 | 86.998 |
| **Pes #5** | 49.098 | - | 24.379 | 31.122 | 33.406 | 28.377 | - | 46.393 | 35.119 | 19.818 | 3.701 | 12.402 | - | 16.103 | 35.921 | - |
| **Mean Manus tracks** | 34.692 | 23.920 | 11.167 | 15.375 | 20.814 | 20.111 | 12.793 | 25.301 | 19.965 | 18.047 | 16.146 | 12.382 | 25.350 | 28.528 | 46.575 | 75.509 |
| **Mean Pes tracks** | 49.609 | 46.895 | 24.682 | 30.187 | 34.636 | 31.826 | - | 48.061 | 35.165 | 15.069 | 11.112 | 12.678 | 57.596 | 23.790 | 39.717 | 87.825 |

**S1 Table.** *(continued)*

| **Trackway**  **S1C Fig** | **Length** | **Width** | **Digit I** | **Digit II** | **Digit III** | **Digit IV** | **Digit V** | **Length I-IV** | **Width I-IV** | **Div. I-II** | **Div. II-III** | **Div. III-IV** | **Div. IV-V** | **Div. II-IV** | **Div. I-IV** | **Div. I-V** |
| --- | --- | --- | --- | --- | --- | --- | --- | --- | --- | --- | --- | --- | --- | --- | --- | --- |
| **Manus #1** | 27.027 | 22.984 | **9.68** | 12.341 | 17.082 | 14.576 | **10.476** | 23.095 | 15.161 | 21.263 | 14.489 | 11.354 | 40.577 | 25.843 | 47.106 | 87.683 |
| **Manus #2** | 31.219 | 24.77 | 9.318 | 10.712 | 16.034 | 14.046 | 11.916 | 21.408 | 18.353 | 14.129 | 11.774 | 15.038 | 49.821 | 26.812 | 40.941 | 90.762 |
| **Pes #2** | **50.372** | - | 19.606 | **22.462** | **25.851** | **24.595** | - | 41.775 | 33.136 | 7.715 | 13.38 | 10.651 | - | 24.031 | 31.746 | - |
| **Manus #3** | 38.848 | 23.300 | 8.988 | 13.560 | 21.312 | 20.754 | 12.995 | 32.555 | 16.609 | 19.569 | 15.436 | 12.508 | 59.403 | 27.944 | 47.513 | 106.916 |
| **Manus #1bis** | 30.626 | 20.494 | 8.903 | 11.865 | 16.855 | 15.883 | 12.149 | 22.475 | 14.568 | 19.339 | 17.449 | 12.174 | 23.527 | 29.623 | 48.962 | 72.489 |
| **Mean Manus tracks** | 31.930 | 22.887 | 9.222 | 12.120 | 17.821 | 16.315 | 11.884 | 24.883 | 16.173 | 18.575 | 14.787 | 12.769 | 43.332 | 27.556 | 46.131 | 89.463 |
| **Mean Pes tracks** | **50.372** | - | 19.606 | **22.462** | **25.851** | **24.595** | - | 41.775 | 33.136 | 7.715 | 13.38 | 10.651 | - | 24.031 | 31.746 | - |

**S1 Table.** *(continued)*

| **Trackway**  **S1D Fig** | **Length** | **Width** | **Digit I** | **Digit II** | **Digit III** | **Digit IV** | **Digit V** | **Length I-IV** | **Width I-IV** | **Div. I-II** | **Div. II-III** | **Div. III-IV** | **Div. IV-V** | **Div. II-IV** | **Div. I-IV** | **Div. I-V** |
| --- | --- | --- | --- | --- | --- | --- | --- | --- | --- | --- | --- | --- | --- | --- | --- | --- |
| **Manus #1** | **13.724** | 13.032 | 3.653 | 4.885 | - | 7.979 | 4.891 | **12.09** | 8.321 | 18.077 | - | - | 83.372 | 40.771 | 58.848 | 142.22 |
| **Manus #3** | 13.975 | 12.153 | 3.845 | 4.33 | 6.199 | 5.347 | 4.481 | 9.579 | 9.459 | 25.903 | 12.461 | 21.537 | 68.199 | 33.998 | 59.901 | 128.1 |
| **Mean Manus tracks** | 13.850 | 12.593 | 3.749 | 4.608 | 6.199 | 6.663 | 4.686 | 10.835 | 8.890 | 21.990 | 12.461 | 21.537 | 75.786 | 37.385 | 59.375 | 135.160 |

**S1 Table.** *(continued)*

| **Tracks** | **Length** | **Width** | **Digit I** | **Digit II** | **Digit III** | **Digit IV** | **Digit V** | **Length I-IV** | **Width I-IV** | **Div. I-II** | **Div. II-III** | **Div. III-IV** | **Div. IV-V** | **Div. II-IV** | **Div. I-IV** | **Div. I-V** |
| --- | --- | --- | --- | --- | --- | --- | --- | --- | --- | --- | --- | --- | --- | --- | --- | --- |
| **Total Mean Manus tracks** | 37.167 | 24.088 | 13.190 | 17.425 | 22.953 | 21.014 | 12.447 | 31.248 | 22.867 | 20.748 | 15.731 | 11.883 | 33.477 | 27.494 | 48.606 | 84.424 |
| **Total Mean Pes tracks** | 45.453 | 29.989 | 18.147 | 22.988 | 27.475 | 25.215 | 15.170* | 38.602 | 29.657 | 14.348 | 13.240 | 12.744 | 33.768 | 25.941 | 40.177 | 71.865 |

* The mean length of the pedal digit V is not representative, as it is observed in few tracks and usually only preserved by the tip impression.
