# Supplementary material for: An archosauromorph dominated ichnoassemblage in fluvial settings from the late Early Triassic of the Catalan Pyrenees (NE Iberian Peninsula)
Source: PLoS One. 2017 Apr 19;12(4):e0174693. doi: 10.1371/journal.pone.0174693 (PMC5396874; doi:10.1371/journal.pone.0174693)
Supplement: S2 Table — (DOCX) [file pone.0174693.s003.docx]

**An Archosauromorph Dominated Ichnoassemblage in Fluvial Settings from the late Early Triassic of the Catalan Pyrenees (NE Iberian Peninsula)** Eudald Mujal*, Josep Fortuny, Arnau Bolet, Oriol Oms, José Ángel López *Corresponding author: Departament de Geologia, Universitat Autònoma de Barcelona, E-08193 Bellaterra, Spain; e-mail: eudald.mujal@gmail.com

**S2 Table. Trackway measurements of *Prorotodactylus mesaxonichnus* isp. nov.** Values resumed in Table 2

| **Trackway holotype (IPS-93870) Fig 5A** |  |  |  |  |  |  |  |  |
| --- | --- | --- | --- | --- | --- | --- | --- | --- |
| **Stride manus** | 256.043 | 314.023 | 323.778 | 308.696 | 325.867 | - | 240.899 |  |
| **Stride pes** | 260.468 | 291.34 | 320.193 | 309.45 | 296.639 | - | 242.538 |  |
| **Pace manus** | 166.452 | 152.423 | 214.443 | 174.315 | 207.938 | 180.049 |  |  |
| **Pace pes** | 269.712 | 254.260 | 271.193 | 260.609 | 253.776 | 232.863 |  |  |
| **Pace angulation manus** | 106.781 | 117.29 | 112.257 | 108.446 | 112.653 | - |  |  |
| **Pace angulation pes** | 59.265 | 66.441 | 74.224 | 74.078 | 74.333 | - |  |  |
| **Width pace manus** | 95.123 | 93.69 | 106.054 | 112.726 | 106.041 |  |  |  |
| **Width pace pes** | 228.362 | 218.396 | 212.079 | 204.439 | 192.592 |  |  |  |
| **Manus-Pes distance** | 53.977 | 84.709 | 50.052 | 76.328 | 40.045 | 67.693 | 64.608 | 65.002 |
| **Div. manus midline** | 14.831 | 9.155 | 17.521 | 12.268 | -3.859 | -7.475 | - | - |
| **Div. pes midline** | 46.572 | 47.397 | 52.535 | 52.902 | 44.094 | 62.94 | - | - |
| **Div. manus-pes digits III** | 31.741 | 38.242 | 35.014 | 40.634 | 47.953 | 70.415 | 24.417 | 18.468 |
| **Glenoacetabular distance** | 156.543 | 154.809 | 200.522 | 182.864 | 196.067 |  |  |  |
| **Glenoacetabular Standard deviation** | 21.537 |  |  |  |  |  |  |  |

Units in mm and degrees.

**S2 Table.** *(continued)*

| **Trackway paratype (IPS-93867) Fig 6A** |  |  |  |  |  |  |  |  |  |
| --- | --- | --- | --- | --- | --- | --- | --- | --- | --- |
| **Stride manus** | 136.043 | 117.197 | 111.625 | 153.426 | 148.331 | - | 149.612 |  |  |
| **Stride pes** | 102.905 | 141.093 | 121.171 | 146.669 | 153.612 | 169.545 | 136.078 |  |  |
| **Pace manus** | 78.110 | 107.508 | 61.358 | 105.130 | 74.244 | 112.630 | - | - |  |
| **Pace pes** | 155.670 | 158.794 | 134.939 | 144.739 | 133.887 | 163.316 | 176.635 | 187.661 |  |
| **Pace angulation manus** | 94.485 | 81.448 | 77.857 | 116.101 | 105.201 | - | - |  |  |
| **Pace angulation pes** | 37.616 | 56.479 | 51.592 | 63.459 | 60.994 | 60.640 | 43.279 |  |  |
| **Width pace manus** | 63.435 | 65.720 | 66.249 | 49.407 | 59.459 | - | - |  |  |
| **Width pace pes** | 147.819 | 130.345 | 125.928 | 118.442 | 127.795 | 147.545 | 169.416 |  |  |
| **Manus-Pes distance** | 42.101 | 60.363 | 55.637 | 37.661 | 53.517 | 36.200 | 50.199 | - | 62.543 |
| **Div. manus midline** | -3.270 | 6.567 | -18.464 | 0.000 | -1.121 | -1.162 | -0.181 | - | -11.180 |
| **Div. pes midline** | 47.070 | 53.555 | 38.547 | - | 62.814 | 45.220 | 37.601 | - | 44.075 |
| **Div. manus-pes digits III** | 43.800 | 60.122 | 20.083 | - | 61.693 | 44.058 | 37.420 | - | 32.895 |
| **Glenoacetabular distance** | 94.787 | 94.054 | 89.679 | 99.378 | 104.303 |  |  |  |  |
| **Glenoacetabular Standard deviation** | 5.581 |  |  |  |  |  |  |  |  |

**S2 Table.** *(continued)*

| **Trackway S1A Fig** |  |  |  |  |  |  |
| --- | --- | --- | --- | --- | --- | --- |
| **Stride manus** | 277.587 | 276.801 | 274.572 | 280.653 |  |  |
| **Stride pes** | 259.488 | 256.344 | 277.843 | 276.149 |  |  |
| **Pace manus** | 148.569 | 182.773 | 139.242 | 172.691 | 140.794 |  |
| **Pace pes** | 197.711 | 236.796 | 198.773 | 226.557 | 161.092 |  |
| **Pace angulation manus** | 114.470 | 116.945 | 121.398 | 127.171 |  |  |
| **Pace angulation pes** | 73.122 | 70.986 | 80.858 | 89.832 |  |  |
| **Width pace manus** | 88.442 | 82.680 | 74.326 | 70.109 |  |  |
| **Width pace pes** | 172.876 | 173.996 | 160.244 | 133.029 |  |  |
| **Manus-Pes distance** | 48.511 | 39.413 | 54.635 | 50.470 | - | 39.956 |
| **Div. manus midline** | -6.683 | 0.000 | 11.927 | 2.131 | 3.455 | 7.676 |
| **Div. pes midline** | 20.729 | - | - | 33.257 | - | 37.838 |
| **Div. manus-pes digits III** | 27.412 | - | - | 31.126 | - | 30.162 |
| **Glenoacetabular distance** | 138.942 | 159.272 | 147.733 | 137.517 |  |  |
| **Glenoacetabular Standard deviation** | 10.014 |  |  |  |  |  |

**S2 Table.** *(continued)*

| **Trackway S1B Fig** |  |  |  |  |  |
| --- | --- | --- | --- | --- | --- |
| **Stride manus** | 244.694 | 255.025 | 250.344 |  |  |
| **Stride pes** | 242.712 | 254.466 | 253.828 |  |  |
| **Pace manus** | 119.644 | 165.706 | 129.415 | 162.386 |  |
| **Pace pes** | 210.140 | 243.842 | 214.303 | 247.625 |  |
| **Pace angulation manus** | 118.144 | 118.952 | 117.760 |  |  |
| **Pace angulation pes** | 64.519 | 67.445 | 66.775 |  |  |
| **Width pace manus** | 71.813 | 73.088 | 74.321 |  |  |
| **Width pace pes** | 189.522 | 189.033 | 190.774 |  |  |
| **Manus-Pes distance** | 61.841 | 64.132 | 52.523 | 70.071 | 50.362 |
| **Div. manus midline** | 0.000 | 0.000 | 0.000 | -6.084 | 9.227 |
| **Div. pes midline** | 56.867 | - | 52.536 | 46.399 | 41.330 |
| **Div. manus-pes digits III** | 56.867 | - | 52.536 | 52.483 | 32.103 |
| **Glenoacetabular distance** | 136.469 | 139.476 | 147.250 |  |  |
| **Glenoacetabular Standard deviation** | 5.563 |  |  |  |  |

**S2 Table.** *(continued)*

| **Trackway S1C Fig** |  |  |  |
| --- | --- | --- | --- |
| **Stride manus** | 374.792 |  |  |
| **Stride pes** | - |  |  |
| **Pace manus** | 232.552 | 172.288 |  |
| **Pace pes** | - | - |  |
| **Pace angulation manus** | 135.020 |  |  |
| **Pace angulation pes** | - |  |  |
| **Width pace manus** | 75.688 |  |  |
| **Width pace pes** | - |  |  |
| **Manus-Pes distance** | - | 49.782 | - |
| **Div. manus midline** | 0.000 | -19.887 | 0.000 |
| **Div. pes midline** | - | 72.552 | - |
| **Div. manus-pes digits III** | - | 92.439 | - |

**S2 Table.** *(continued)*

| **Trackway S1D Fig** |  |  |  |
| --- | --- | --- | --- |
| **Stride manus** | 106.110 |  |  |
| **Stride pes** | 108.859 |  |  |
| **Pace manus** | 76.279 | 68.180 |  |
| **Pace pes** | 99.972 | 65.257 |  |
| **Pace angulation manus** | 94.477 |  |  |
| **Pace angulation pes** | 79.311 |  |  |
| **Width pace manus** | 48.511 |  |  |
| **Width pace pes** | 58.585 |  |  |
| **Manus-Pes distance** | 39.641 | 20.269 | 36.307 |
| **Div. manus midline** | 0.000 | - | 0.000 |
| **Div. pes midline** | - | - | - |
| **Div. manus-pes digits III** | - | - | - |
