# Supplementary figures and images for: An archosauromorph dominated ichnoassemblage in fluvial settings from the late Early Triassic of the Catalan Pyrenees (NE Iberian Peninsula)
Source: PLoS One. 2017 Apr 19;12(4):e0174693. doi: 10.1371/journal.pone.0174693 (PMC5396874; doi:10.1371/journal.pone.0174693)

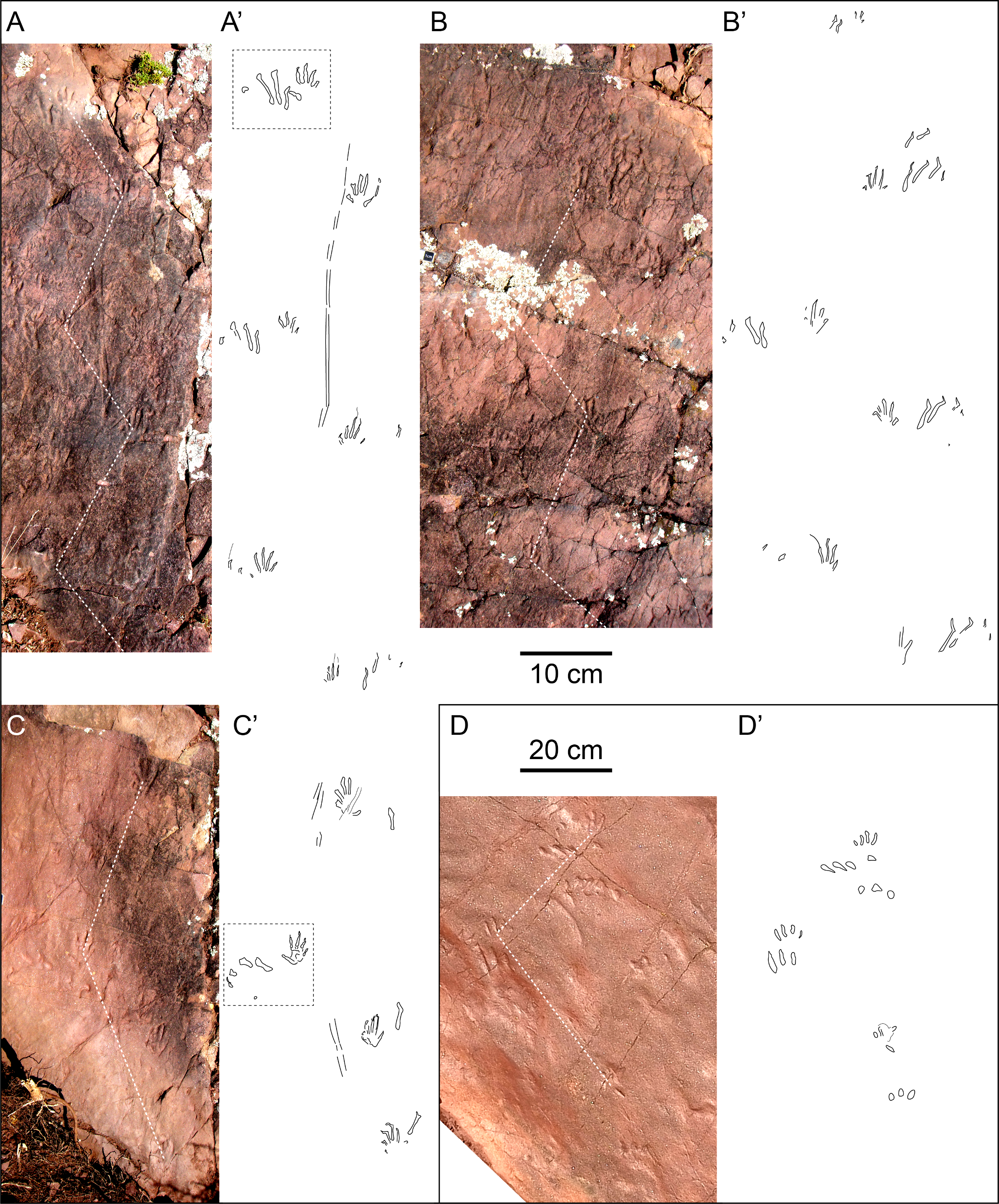

Supplement: S1 Fig — (A-C) Specimens from Erillcastell; dashed squares from A and C are correspond to manus-pes sets from Fig 7A and 7B, respectively. (D) Specimen from Buira. (TIF) [file pone.0174693.s004.tif]

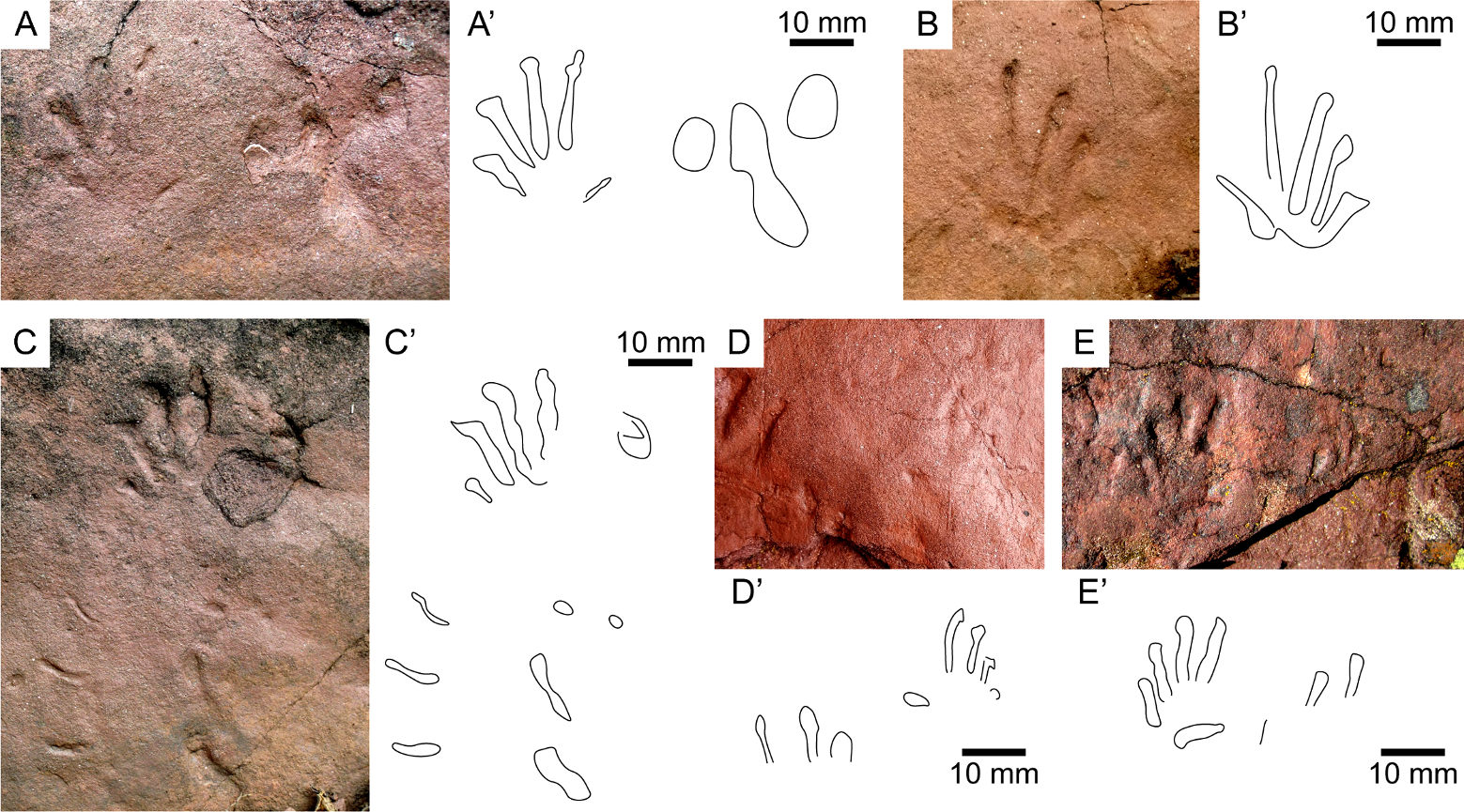

Supplement: S2 Fig — (TIF) [file pone.0174693.s005.tif]

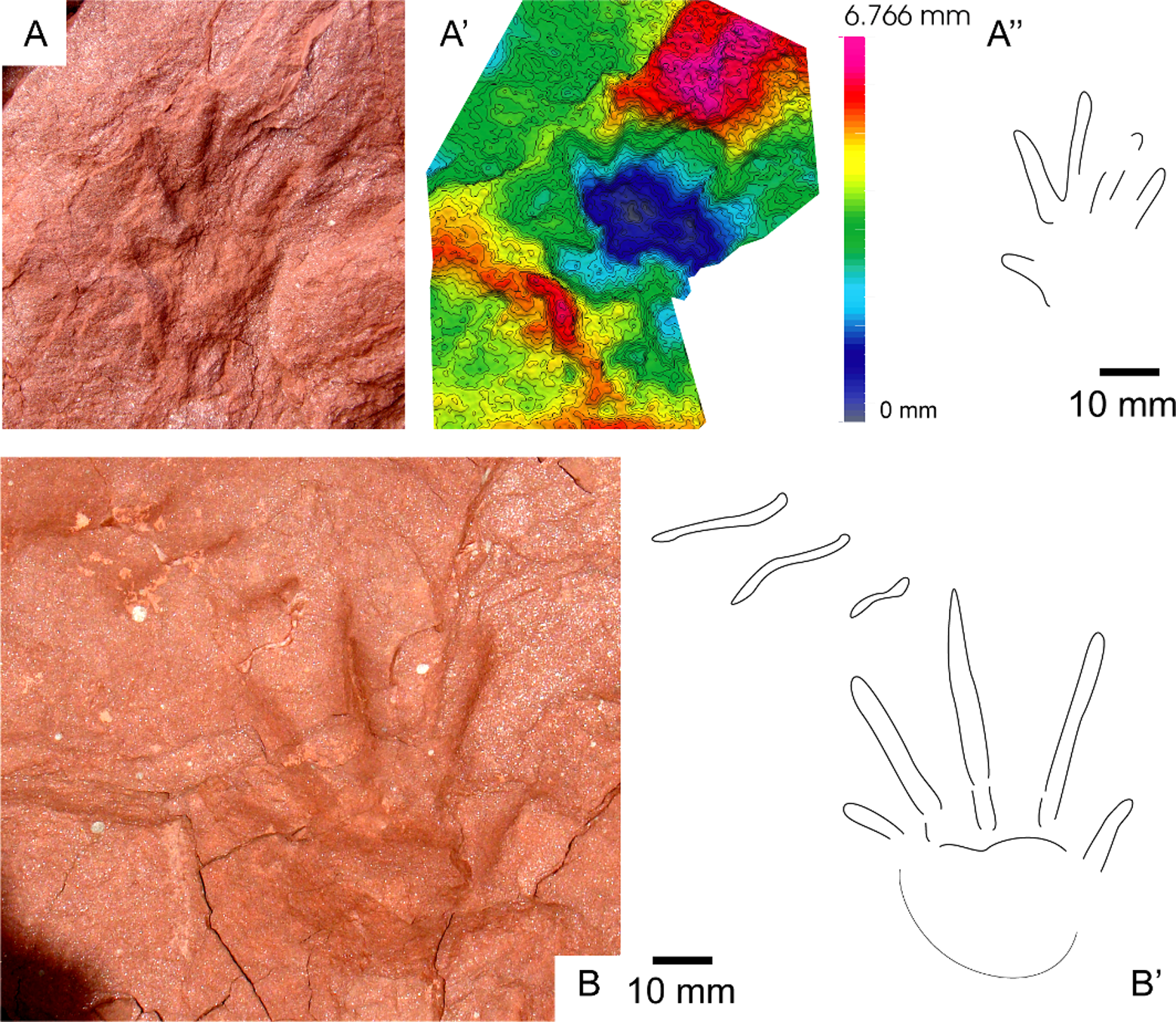

Supplement: S3 Fig — Ichnites from sections IV (A) and VII (B). (TIF) [file pone.0174693.s006.tif]

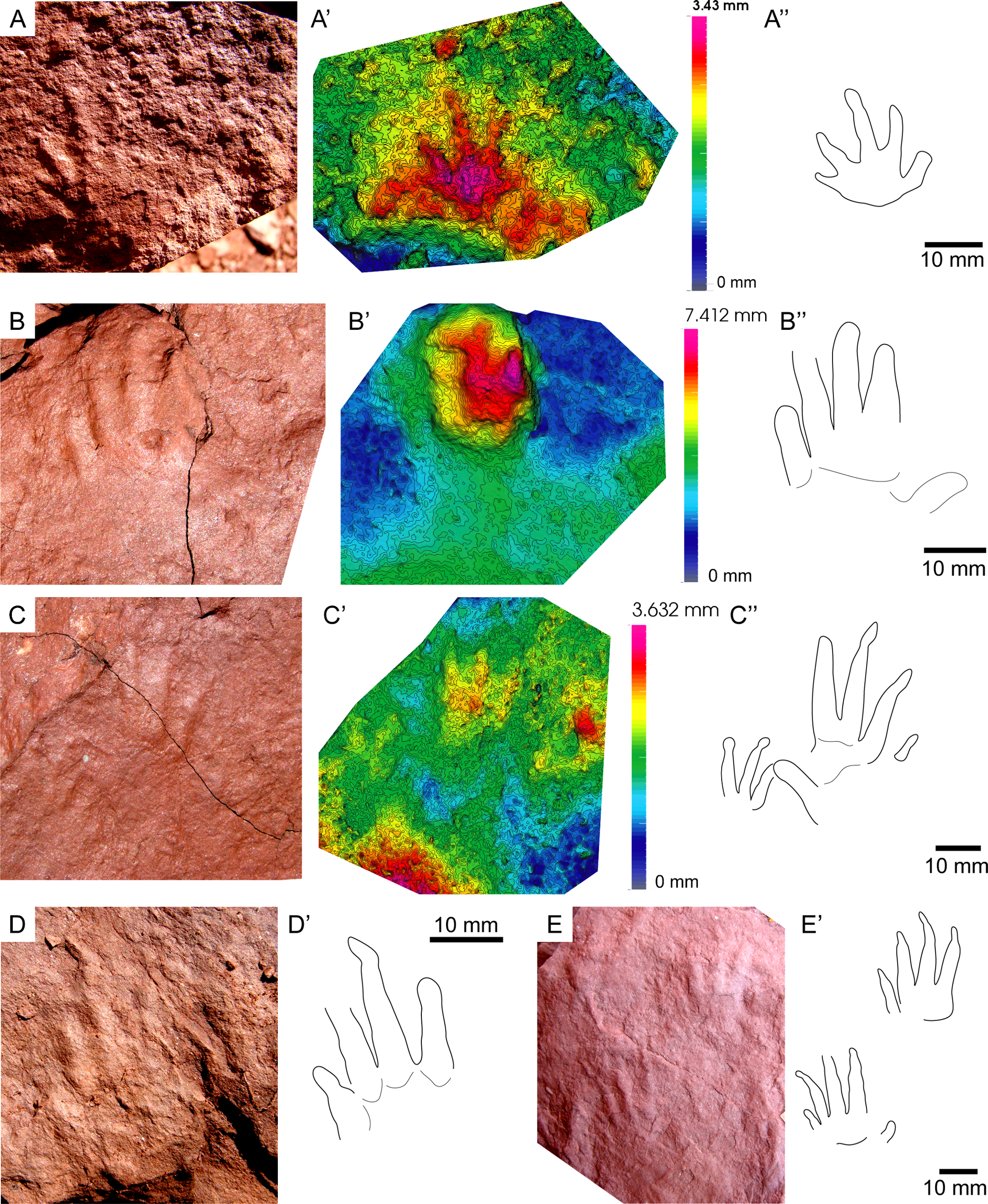

Supplement: S4 Fig — (A) IPS-83740 from Argestues tracksite. (B-E) Isolated ichnites from Rubió tracksite. Note the relatively bad preservation of the ichnites, all in convex hyporelief, preserved in the bases of small meandering channels (facies St). (TIF) [file pone.0174693.s007.tif]

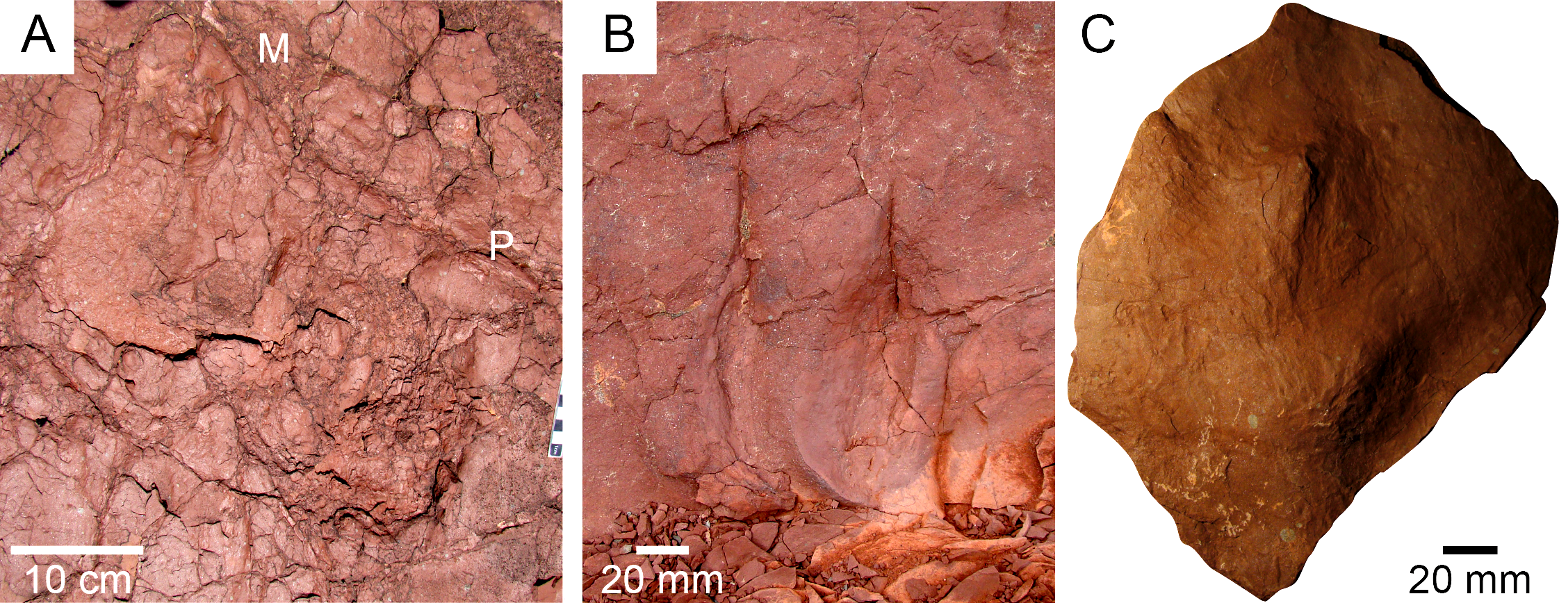

Supplement: S5 Fig — (A) Manus-pes set (M-P). (B) Scratch-like track. (C) Partial track of IPS-83750. (TIF) [file pone.0174693.s008.tif]

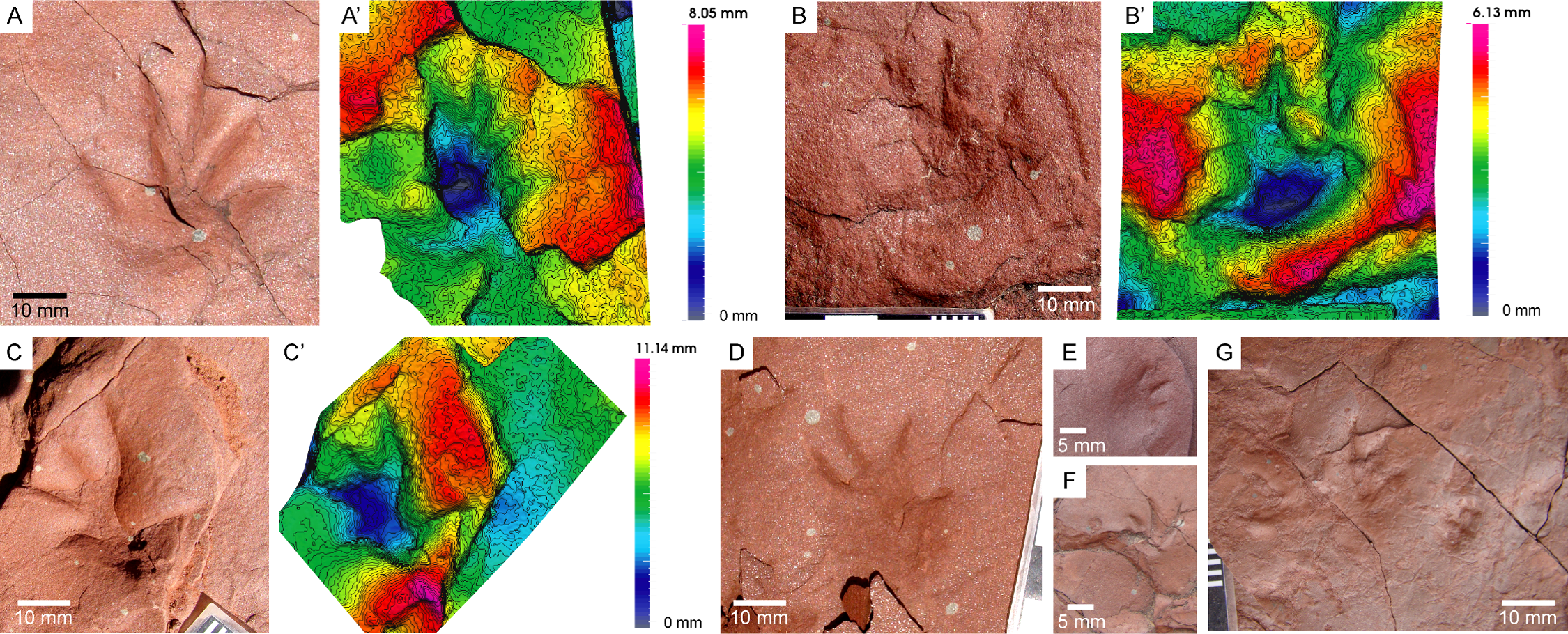

Supplement: S6 Fig — (A-C) Rhynchosauroides cf. schochardti. (D) Rhynchosauroides isp. indet. 1. (E-G) Rhynchosauroides isp. indet. (TIF) [file pone.0174693.s009.tif]

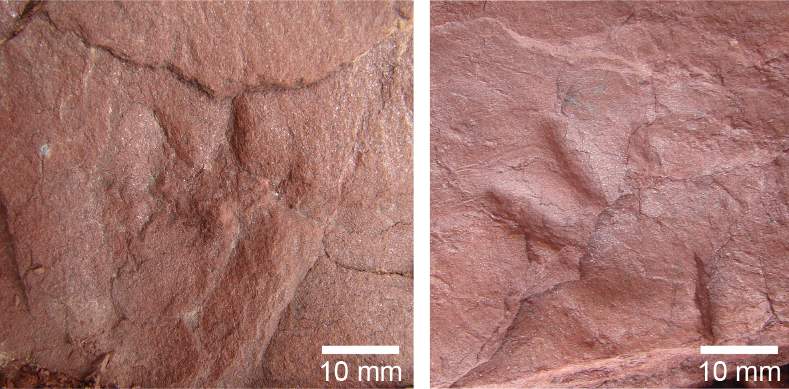

Supplement: S7 Fig — (TIF) [file pone.0174693.s010.tif]
